# Coordination of ethane, pentane and cyclopentane to a cationic osmium complex: comparisons in alkane binding†

**DOI:** 10.1039/d5sc00973a

**Published:** 2025-04-11

**Authors:** James D. Watson, Dejan Mizdrak, Leslie D. Field, Graham E. Ball

**Affiliations:** a School of Chemistry, University of New South Wales Sydney NSW 2052, Australia l.field@unsw.edu.au g.ball@unsw.edu.au

## Abstract

When a solution of [η^5^-CpOs(CO)_3_]^+^[Al(OC(CF_3_)_3_)_4_]^−^ is photolyzed in the presence of ethane, pentane or cyclopentane, photo-liberation of carbon monoxide occurs and the corresponding metal-alkane σ-complex, [η^5^-CpOs(CO)_2_(alkane)]^+^ (where alkane = ethane, pentane and cyclopentane), forms. Here we report the NMR spectroscopic and computational investigations into the structure, reactivity, lifetimes and binding energies of the osmium-centred alkane σ-complexes [η^5^-CpOs(CO)_2_(C_2_H_6_)]^+^, [η^5^-CpOs(CO)_2_(*n*-C_5_H_12_)]^+^ and [η^5^-CpOs(CO)_2_(c-C_5_H_10_)]^+^. The fragment [η^5^-CpOs(CO)_2_]^+^ binds alkanes tightly and forms remarkably stable complexes with ethane, *n*-pentane and cyclopentane. The effective half-life for [η^5^-CpOs(CO)_2_(*n*-C_5_H_12_)]^+^ and [η^5^-CpOs(CO)_2_(c-C_5_H_10_)]^+^ are 0.95 and 0.21 h respectively at −50 °C, making these amongst the most stable metal-alkane complexes in solution reported to date. Different isomers of the *n*-pentane complexes are observed and the relative amount of each in solution is strongly dependent on the presence of photo-irradiation. When irradiated, the methyl-bound (C1) isomer is the major product and in the absence of irradiation the system equilibrates, and the methylene-bound isomers (C2 and C3) are the major products.

## Abstract 2

NMR and DFT investigations into osmium-alkane σ-complexes of pentane, cyclopentane and ethane. The cyclopentane and *n*-pentane complexes are amongst the most stable alkane σ-complexes characterized to date.

## Graphical abstract

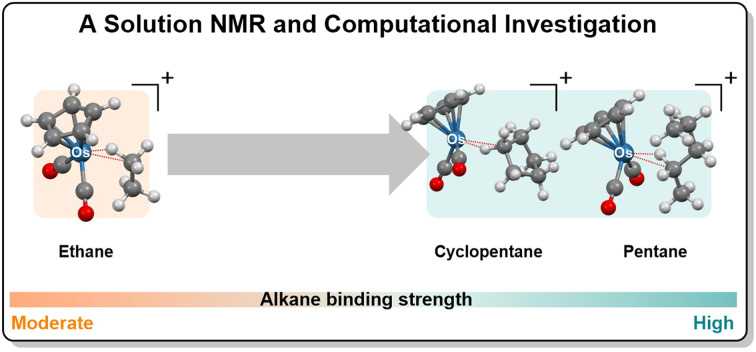

## Introduction

Alkanes are comprised exclusively of C–C and C–H bonds and were historically named as paraffins because of their lack of reactivity.^1,2^ The inertness of alkanes can be attributed to the fact that: (i) carbon and hydrogen atoms have very similar electronegativities. C–H bonds are essentially non-polar and alkanes have a very poor ability to donate electrons; (ii) the presence of three other substituents on the carbon sterically hinders the overlap of orbitals on the metal with those on the carbon, limiting back donation in particular; and (iii) C–C and C–H bonds have relatively large bond dissociation energies (BDEs), typically between 79-90 kcal mol^−1^ and 90–105 kcal mol^−1^ respectively^3^ and the large BDE dictates that C–C and C–H bonds require significant amounts of energy to be broken.^4^ While alkanes are unreactive, they are an abundant, relatively cheap resource and this makes them attractive feedstocks for the synthesis of more complex, more valuable species. For this reason, selective catalytic conversion of alkanes into other products, such as alcohols or olefins has been the subject of intense investigation for several decades.

**Scheme 1:**
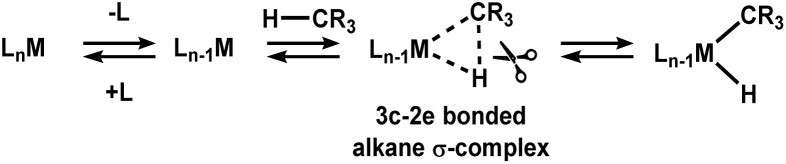
An illustration of how coordinatively unsaturated metal complexes and C–H bonds can interact to form a 3-centre 2-electron (3c-2e) σ-complex prior to the oxidative cleavage of the C–H bond.

Alkane σ-complexes are widely accepted as intermediates in the activation of C–H bonds by many transition metal catalysts *en route* to the oxidative cleavage of a C–H bond to a transition metal centre (Scheme 1).^5^

Alkane σ-complexes involve the 3-centre 2-electron (3c-2e) interaction between a transition metal and the electrons that are occupying the C–H bond orbitals in an alkane.^6^ The very weak nature of the 3c-2e metal-alkane interaction results in very short lifetimes of alkane σ-complexes. The technical limitations of common spectroscopic techniques make detailed interrogation of short-lived alkane σ-complexes exceptionally challenging. Seminal pump-probe, FTIR investigations, initially conducted by Perutz and coworkers, demonstrated that organometallic alkane σ-complexes could be generated by photolysis of metal-carbonyl compounds in the presence of alkanes and could be detected using IR spectroscopy to observe changes in the stretching frequency of the carbonyl ligands.^7–10^ These pump-probe investigations were the first detailed investigations into metal-alkane complexes and provided insights into properties such as species lifetime and alkane binding strength but did not elucidate the binding motif of the alkane to the transition metal.

**Scheme 2:**
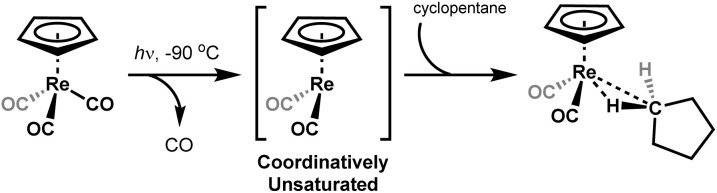
The photolysis of [η^5^-CpRe(CO)_3_] in a cyclopentane solution at −90 °C to generate the alkane complex, [η^5^-CpRe(CO)_2_(c-C_5_H_10_)].^11^

We reported the first NMR spectroscopic observation of a metal-alkane σ-complex when cyclopentadienyl rhenium tricarbonyl was irradiated in a solution of cyclopentane at low temperature inside the probe of an NMR spectrometer (Scheme 2).^11^

We have since developed investigation of metal-alkane σ-complexes, informed by the computational probes of Head-Gordon and coworkers,^12,13^ and we have characterized a series of cationic metal-alkane σ-complexes, such as [η^6^-(HEB)Re(CO)_2_(alkane)]^+^ (where HEB = hexaethylbenzene, alkane = cyclopentane and pentane). These species bind alkanes even more tightly than their neutral isoelectronic analogues and experimentally corroborate the computational investigations.^14^ More recently we reported [η^5^-CpOs(CO)_2_]^+^,^15,16^ which reacts, with methane to form the osmium methane σ-complex, [η^5^-CpOs(CO)_2_(CH_4_)]^+^ at low temperature. We conducted this study in CF_3_CH_2_CF_3_ (which is commercially available in high purity, see ESI† for details) as an inert solvent using the very weakly coordinating “Krossing” anion, [Al(OC(CF_3_)_3_)_4_]^−^,^17–22^ as the counterion to employ “gas-phase like” conditions and minimize any competing binding interactions.^21,23^ Girolami and coworkers have also recently reported low temperature NMR spectroscopic investigations into the similar [η^5^-Cp*Os((CF_3_)_2_PCH_2_P(CF_3_)_2_)(CH_4_)]^+^.^24^ There, they experimentally demonstrate (i) the influence of the supporting phosphine ligand and show that the electron withdrawing π-acid phosphines (*e.g.* (CF_3_)_2_PCH_2_P(CF_3_)_2_) stabilize the methane σ-complex while more electron donating phosphines promote oxidative cleavage of the coordinated C–H bond;^24–26^ and (ii) use isotopic perturbation of equilibrium experiments to determine that the binding mode of the bound methane must be κ^1^-H or η^2^-C,H. Several groups have used solution-NMR spectroscopy to experimentally interrogate the interaction of a variety of linear (C_*n*_H_2*n*+2_, where *n* > 1), and cyclic (C_*n*_H_2*n*_, where *n* > 3) alkanes with a range of isoelectronic reactive organometallic fragments.^14,27–35^ Weller and coworkers have used solid-state organometallic (SMOM) synthesis to produce metal-alkane σ-complexes and have conducted solid-state-NMR (SSNMR) spectroscopic investigations to probe the binding preference of metal centre for the alkane in the solid state.^36–44^ These investigations have shown that generally; (i) methylene groups, –CH_2_–, interact preferentially with the organometallic transition metal complexes than methyl –CH_3_– groups; (ii) cycloalkanes interact more strongly than linear alkanes, and (iii) heavier alkanes interact more strongly with the metal centre than light alkanes.^27–35,45–53^

In this paper, we expand the scope of metal-alkane interactions to probe the interaction of [η^5^-CpOs(CO)_2_]^+^ with ethane, pentane and cyclopentane and we report the NMR spectroscopic and DFT calculations on the structure, characteristics and reactivity of the alkane σ-complexes [η^5^-CpOs(CO)_2_(alkane)]^+^ [where alkane = ethane (2), *n*-pentane (4), and c-pentane (5)].

## Results and discussion

### Coordination of ethane, [η^5^-CpOs(CO)_2_(C_2_H_6_)]^+^ (2)

To date, only two ethane σ-complexes, have been studied using NMR spectroscopy and only one of these species was formed by intermolecular reaction of an active metal fragment with molecular ethane.^35,51^

**Scheme 3:**
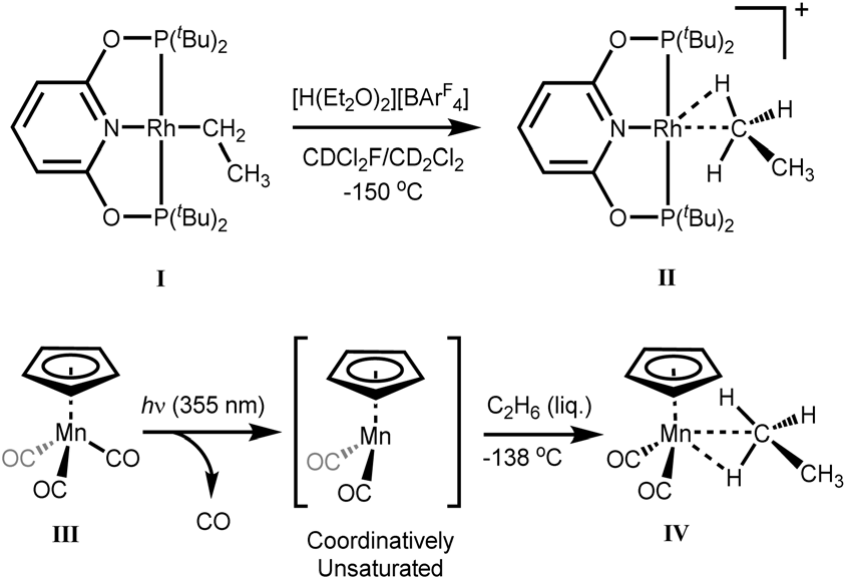
(Top) Protonation of [(PONOP)Rh(C_2_H_5_)] complex with [H(Et_2_O)_2_]^+^[B(Ar^F^)]^−^ in CDCl_2_F/CD_2_Cl_2_ at −150 °C to form the cationic rhodium ethane complex [(PONOP)Rh(C_2_H_6_)]^+^. (Bottom) Photolysis of [η^5^-CpMn(CO)_3_] in high pressures of liquid ethane at −138 °C to generate the ethane complex [η^5^-CpMn(CO)_2_(C_2_H_6_)].

The first ethane σ-complex, [(PONOP)Rh(C_2_H_6_)]^+^(II) (PONOP = κ^3^-NC_5_H_3_-2,6-(OP^*t*^Bu_2_)_2_), was formed by protonation of the neutral Rh–ethyl complex, [(PONOP)Rh(C_2_H_5_)] (I)*in situ* with the strong acid [H(Et_2_O)_2_]^+^[B(Ar^F^)_4_]^−^; [B(Ar^F^)_4_]^−^ = tetrakis[3,5-bis(trifluoromethyl)phenyl]borate)], at low temperatures inside an NMR spectrometer (Scheme 3).^51^ The cationic ethane σ-complex was observed for several hours at −132 °C in a solution of CDCl_2_F.

The second example of an ethane σ-complex for which NMR data has been reported, is the ethane σ-complex [η^5^-CpMn(CO)_2_(C_2_H_6_)]^35^ derived from cyclopentadienyl manganese tricarbonyl. The neutral ethane σ-complex was formed by reaction of molecular ethane with the photolytically-generated reactive metal fragment, [η^5^-CpMn(CO)_2_], and had a reported lifetime of 2.6 μs at room temperature (by IR spectroscopy).^35^ When Perutz and co-workers photolyzed the cyclopentadienyl tricarbonyl precursor (III) under high pressures of liquefied ethane at very low temperatures (−138 °C) they formed the corresponding manganese-ethane σ-complex, [η^5^-CpMn(CO)_2_(C_2_H_6_)] (IV) which had a reported lifetime of ∼6 minutes under the experimental conditions.^35^

Both the rhodium- and manganese-centred ethane σ-complexes have been well examined computationally by Bistoni and co-workers.^18^ Despite their different binding modes and ligand scaffolds, calculations show that the relative stability of both systems can be attributed in part to strong dispersion interactions between the metal centre and the coordinated ethane.^17,18^

**Fig. 1:**
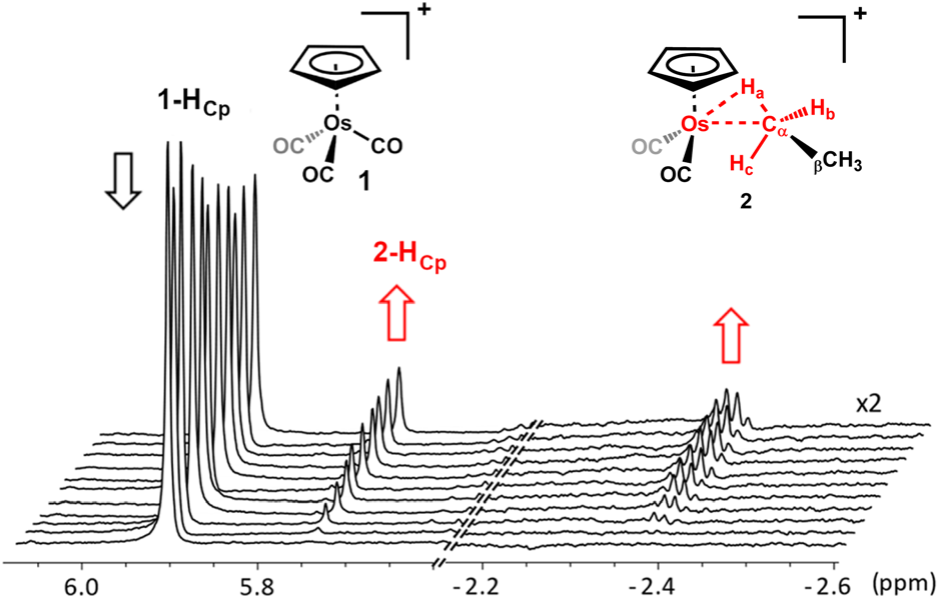
Selected portions of the 600 MHz ^1^H NMR spectra that were collected during photolysis of [η^5^-CpOs(CO)_3_]^+^(1) in an ethane-saturated solution of 1,1,1,3,3,3 hexafluoropropane (HFP) at *−*90 °C. The spectra, collected at ∼2-minute intervals, illustrate the concomitant decrease in the intensity of the Cp resonance for the starting material and increase in intensity of the resonances of the product during photolysis. A rapid exchange process renders the three hydrogens coloured red equivalent, leading to the averaged resonance at *δ* −2.39 (see text below and Scheme 4 for details).

**Fig. 2:**
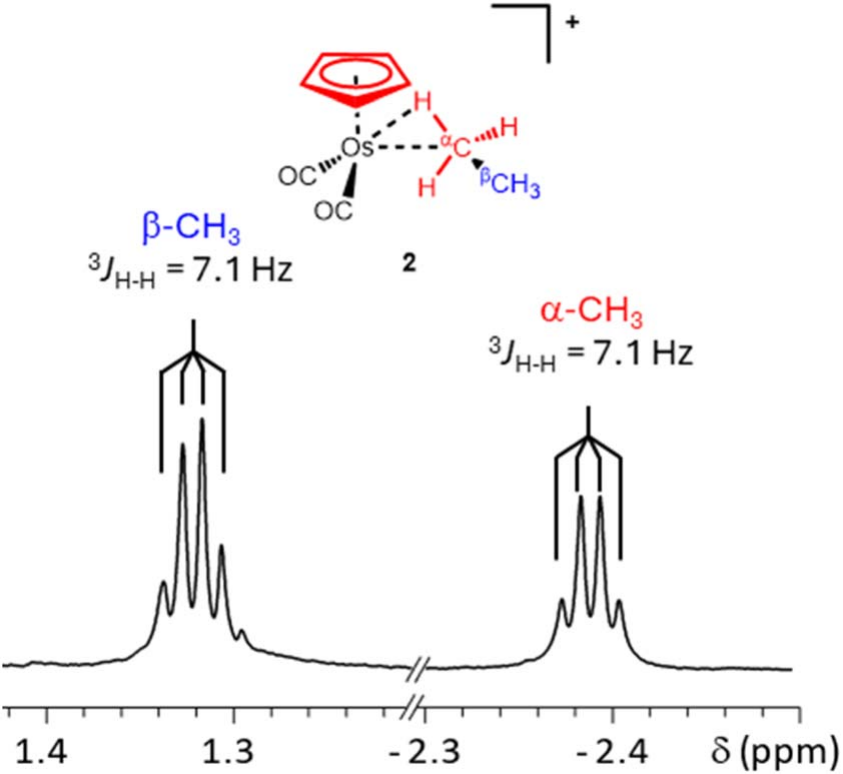
Expansion of the high field and very high field regions of the 700 MHz ^1^H NMR spectrum (in CF_3_CH_2_CF_3_ solvent at −90 °C) of the osmium ethane complex [η^5^-CpOs(CO)_2_(C_2_H_6_)]^+^(2) illustrating the ^3^*J*_H–H_ coupling between the bound and non-bound CH_3_ groups.

When a solution of [η^5^-CpOs(CO)_3_]^+^(1) in 1,1,1,3,3,3-hexafluoropropane (HFP) saturated with ethane is photolyzed (100 W, Hg arc lamp) at −90 °C, the resonances characteristic of [η^5^-CpOs(CO)_2_(C_2_H_6_)]^+^(2) become visible in the ^1^H NMR spectrum. While it is likely that the HFP complex forms during photolysis of the precursor complex, [η^5^-CpOs(CO)_3_]^+^(1) and there is likely an equilibrium amount of this complex present, we have made no spectroscopic observation of this species throughout our investigations here or previously.^15,16^ In the ^1^H NMR spectrum, a quartet resonance at high field (*δ* −2.39) grows in intensity as the photolysis progresses. A resonance in the Cp region (*δ* 5.75) and a quartet resonance in the alkyl region (*δ* 1.32) also grow as the resonance corresponding to the starting material diminishes (Fig. 1 and 2).

As we have stated previously,^15^ when (1) is photolyzed in alkane/HFP mixed solvent systems at low temperatures, several resonances, which are not ascribed to [η^5^-CpOs(CO)_2_(alkane)]^+^, grow in the Cp region of the ^1^H NMR spectrum. The yield of these species is variable, and we have not observed any other resonances in the ^1^H NMR spectra that we can assign to these species. At this time, we have not conclusively identified these species but it is possible that they are dimers derived from 1, similar to the rhenium complexes such as [Re_2_Cp_2_(CO)_5_] proposed as the terminal decomposition product formed when [η^5^-CpRe(CO)_3_] is irradiated or complexes of very low concentrations of impurities present in the solvent. Aside from crowding the Cp region of the ^1^H NMR spectrum, these species do not materially interfere with our analyses.

During photolysis, the resonances for [η^5^-CpOs(CO)_2_(C_2_H_6_)]^+^(2) grow in the ^1^H NMR spectrum, typically reaching a maximum intensity (yield *ca.* 20%) after ∼20 minutes. When the UV lamp is switched off, the complex is relatively stable at −90 °C and can be observed for many hours at this temperature, enabling detailed analysis by NMR spectroscopy. The decay of the ethane complex [η^5^-CpOs(CO)_2_(C_2_H_6_)]^+^(2), at a slightly higher temperature of −84 °C can be fitted to a first order process which gives an effective half-life of approximately 14 h (ESI Graph S1 and Table S1†). While care should be taken comparing the decay rates of different alkane complexes in different conditions and in different samples, this means that the half-life of the osmium ethane complex, 2, at −84 °C is slightly longer than the analogous methane complex, [η^5^-CpOs(CO)_2_(CH_4_)]^+^ measured at a lower temperature (*t*_1/2_ = 13 h at −90 °C).^15^ The lifetime of (2) at −84 °C is approximately five times longer than the half-life reported for the rhodium ethane complex, [(PONOP)Rh(C_2_H_6_)]^+^, (*t*_1/2_ = 5.5 h at −132 °C)^35,51^ and almost 300 times longer than the manganese–ethane complex, [η^5^-CpMn(CO)_2_(C_2_H_6_)] (*t*_1/2_ = 360 s at −138 °C).^35^

The high field resonance at *δ* −2.39 and the resonance at *δ* 1.32 (Fig. 2) appear as proton-coupled quartets (^3^*J*_H–H_ = 7.1 Hz) and are assigned to the metal-bound (*α*) protons and the non-bound (*β*) protons of the osmium-bound ethane respectively. The quartet multiplicity, which arises from the CH_3_– to –CH_3_ coupling, was established through selective decoupling experiments and these experiments conclusively assign the resonances at *δ* 1.32 and *δ* −2.39 to the osmium-bound ethane.

The stability of the ethane complex, 2, was also measured at −76 °C in a different sample. At this temperature, the decay of the Cp resonance was used to calculate the effective half-life of 2 at −76 °C (∼35 minutes – ESI Graph S2 and Table S2†). The ^1^H resonances of 2 at *δ* 1.32 and *δ* −2.39 were correlated to ^13^C resonances at *δ* 11.1 and *δ* −30.7 respectively using heteronuclear single quantum coherence (HSQC) NMR experiments (ESI Fig. S2 and S3†). Both the ^1^H and ^13^C chemical shifts of the osmium-bound methyl group of the complexed ethane (*α*-CH_3_) are significantly more shielded than the ^1^H and ^13^C resonances of free ethane (*δ*^1^H = 0.84 and *δ*^13^C = 5.3) and the ^1^H and ^13^C chemical shifts of the methyl which is not bound to the osmium centre (β-CH_3_) are slightly less shielded than the resonances of free ethane.

When calculated at the same level of theory (DLPNO-CCSD(T1)/def2-QZVPP//MN15/def2TZVP) used for the previously reported osmium methane complex, the *binding free energy* for the interaction of the cationic, reactive osmium metal fragment, [η^5^-CpOs(CO)_2_]^+^ with ethane was −16.6 kcal mol^−1^ and this value is 2.7 kcal mol^−1^ lower in energy (*i.e.* a stronger interaction) than in the analogous interaction in the methane complex, [η^5^-CpOs(CO)_2_(CH_4_)]^+^ (details in ESI†; see also Table 1). This calculation supports the experimental observation that the ethane complex is more stable (*i.e.* it has a longer half-life) than the corresponding methane complex at the same temperature.

**Table 1:** Calculated binding energies and binding energies relative to the binding of methane of different alkanes to [CpOs(CO)_2_]^+^. Details of all calculations in the ESI

Alkane	Binding energy (kcal mol^−1^)		Binding energy relative to methane^c^ (kcal mol^−1^)		
	Electronic energy^a^	Gibbs free energy in the gas phase^b^	Electronic energy	Gibbs free energy in the gas phase	Gibbs free energy in CF_3_CH_2_CF_3_ solvent^d^
Methane	−20.3	−14.3	0	0	0
Ethane	−23.8	−17.3	−3.5	−3.0	−1.7
Cyclopentane^e^	−28.8	−21.7	−8.5	−7.4	−3.9
*n*-Pentane-C1	−26.4	−18.9	−6.1	−4.6	−2.1
*n*-Pentane-C2	−28.0	−20.8	−7.8	−6.5	−3.2
*n*-Pentane-C3	−28.6	−21.2	−8.3	−6.9	−3.2

a Calculated at the DLPNO-CCSD(T1)/CBS//TPSS-D4/def2-TZVP level of theory.

b Gibbs free energies calculated with corrections to the electronic energy calculated using TPSS-D4/def2-TZVP. These corrections are calculated using a single conformation and do not account for the number of possible configurations of alkane binding. Gibbs free energies calculated at 173 K at 1 atm of pressure.

c Relative energies are for the reaction [CpOs(CO)_2_(CH_4_)]^+^ + alkane → [CpOs(CO)_2_(alkane)]^+^ + CH_4_.

d Solvation energies calculated using the SMD model with M05-2X/6-31+G(d,p). Absolute binding energies in solvent are not calculated, only relative binding energies.

e Free cyclopentane is assumed to have a rotational symmetry number of 10 in these calculations due to the fast pseudorotation of the cyclopentane ring leading to effectively a *D*_5h_ point group.^54^

**Scheme 4:**
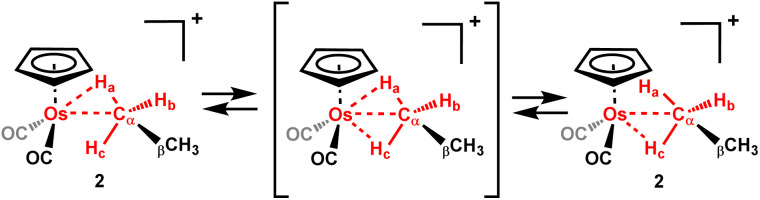
The rapid bond interchange between the hydrogens (H–H bond swap) in the coordinated CH_3_ unit in [η^5^-CpOs(CO)_2_(C_2_H_6_)]^+^(2) – the calculated barrier to exchange is about 4 kcal mol^−1^.

In the ^1^H NMR spectrum of the osmium–ethane complex, [η^5^-CpOs(CO)_2_(C_2_H_6_)]^+^(2), the 3 hydrogens of the metal-bound CH_3_ group are in rapid exchange on the NMR timescale at −90 °C. Calculations predict a barrier of approximately 4 kcal mol^−1^ for the H–H bond-swap (Scheme 4). The exchange of hydrogens of a bound methylene or methyl group directly interacting with the metal centre has been fast on the NMR timescale, in all alkane complexes observed using NMR spectroscopy to date.^14,27,28,31,33–35,55,56^

**Scheme 5:**
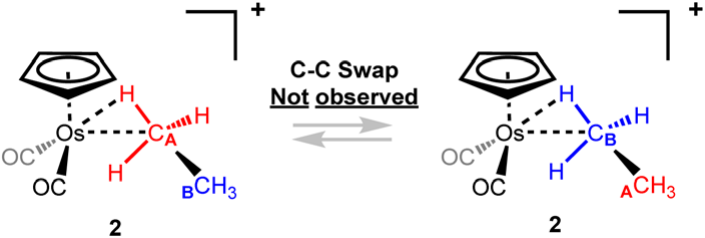
C–C swapping between the bound and non-bound CH_3_ groups of the coordinated ethane is not observed at −84 °C.

No exchange has been observed between the bound and non-bound methyl groups of the complexed ethane in [η^5^-CpOs(CO)_2_(C_2_H_6_)]^+^(2) at −84 °C in selective 1D ^1^H EXSY NMR experiments^14^ (making the upper limit for exchange < 0.1 s^−1^ at this temperature). In contrast, the rhodium complex, [(PONOP)Rh(C_2_H_6_)]^+^(II), reported by Brookhart and co-workers,^51^ the α and β carbons of the metal-bound ethane exchange readily, and NMR evidence indicates that the barrier to exchange is about 7.2 kcal mol^−1^ at −132 °C.^51^ [η^5^-CpOs(CO)_2_(C_2_H_6_)]^+^. (2), has a significantly higher barrier to C–C swapping (13.7 kcal mol^−1^ calculated) than the rhodium–ethane complex, [(PONOP)Rh(C_2_H_6_)]^+^ (Scheme 5 and ESI Fig. S10†).

**Fig. 3:**
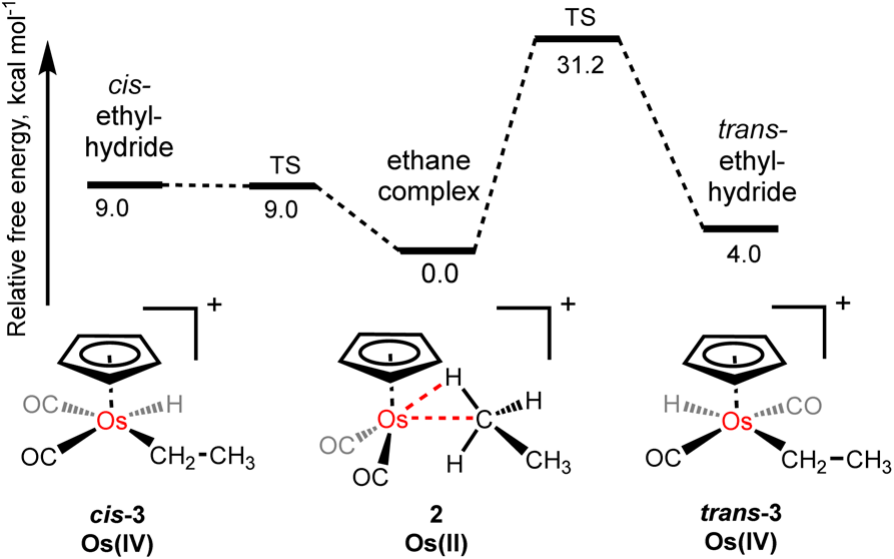
The calculated relative energies (and barriers) to oxidative cleavage of coordinated C–H bond in [η^5^-CpOs(CO)_2_(C_2_H_6_)]^+^(2) to form *cis-* or *trans-*ethyl hydrides.

Throughout the NMR investigations of the osmium–ethane complex, [η^5^-CpOs(CO)_2_(C_2_H_6_)]^+^(2), we have seen no evidence of oxidative cleavage of a C–H bond to form either the corresponding *cis*-osmium(iv) ethyl hydride complex (*cis*-3), or the *trans*-osmium(iv) ethyl hydride complex (*trans*-3). This is likely due to the π-acidity of the carbonyl supporting ligands and computational modelling of the oxidative cleavage of the coordinated C–H bond suggests that the barrier to form the *cis*-osmium(IV) ethyl hydride complex (*cis*-3) from the σ-ethane complex (2), is approximately 9.0 kcal mol^−1^ and the *cis*-ethyl hydride complex is approximately 9.0 kcal mol^−1^ higher in energy than the σ-bound ethane complex. Interestingly, the fact that these two energies are essentially the same implies the conversion of the *cis*-ethyl hydride complex to the ethane complex is barrierless in this case. Although we cannot experimentally rule out a situation where there is an equilibration between ethyl hydride complex (*cis*-3) from the σ-ethane complex (2), that is fast on the NMR timescale even at −90 °C, the calculated difference in energy suggests the equilibrium amount of alkyl hydride would be negligible. While the *trans*-ethyl hydride complex (*trans*-3) is calculated to be just 4.0 kcal mol^−1^ higher in energy than the σ-bound ethane complex, 2, there is a high barrier to form this species (approximately 31.2 kcal mol^−1^) (Fig. 3).

### Coordination of pentane, [η^5^-CpOs(CO)_2_(*n*-C_5_H_12_)]^+^ (4)

**Fig. 4:**
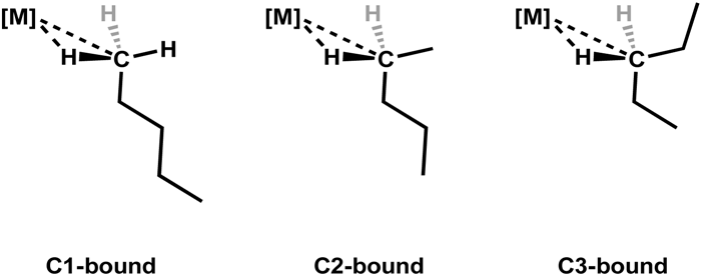
The possible positional isomers for *n*-pentane bound to a metal complex.^14,27,33^

Previous solution-state investigations into *n*-pentane σ-complexes have been conducted on the rhenium and tungsten metal centres, [η^5^-CpRe(CO)_2_(*n*-C_5_H_12_)],^27^ [η^6^-(HEB)W(CO)_2_(*n*-C_5_H_12_)],^33^ and [η^6^-(HEB)Re(CO)_2_(*n*-C_5_H_12_)]^+^.^14^ These investigations involved generation of the *n*-pentane complexes by *in situ* photolysis of the corresponding tricarbonyl precursor in the presence of pentane. The pentane can be bound to the metal *via* C1, C2 or C3 of the pentane chain (Fig. 4). In the case of the neutral rhenium *n*-pentane σ-complex, [η^5^-CpRe(CO)_2_(*n*-C_5_H_12_)], there is a slight preference for binding to the methylene, groups over the methyl groups, whereas the HEB bearing complexes, [η^6^-(HEB)W(CO)_2_(*n*-C_5_H_12_)] and [η^6^-(HEB)Re(CO)_2_(*n*-C_5_H_12_)]^+^ exhibit a clear preference for binding at the methyl groups over the methylene groups. It was proposed that the preference for binding methyl groups in the complexes with HEB ligands was probably due to the increased steric demand of the ethyl substituents on the bulkier HEB ligand.^14,27,33,57–60^

**Fig. 5:**
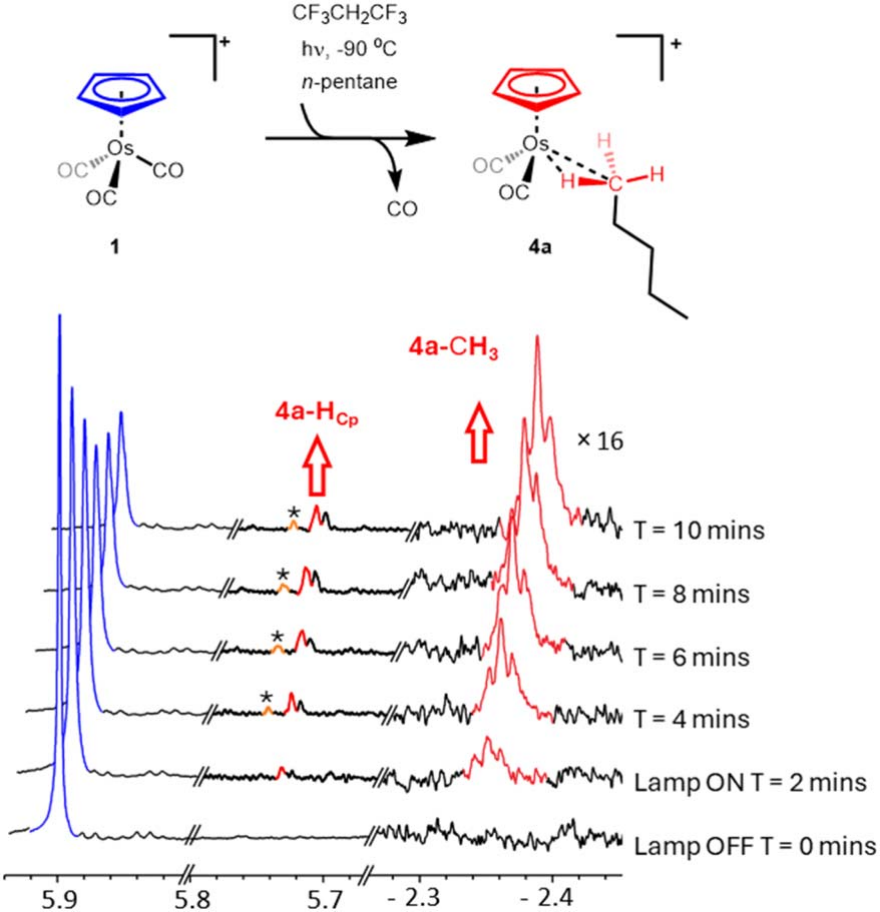
Selected regions of the 700 MHz ^1^H NMR spectra collected during irradiation of [η^5^-CpOs(CO)_3_]^+^ (1) in a mixed solvent of 1,1,1,3,3,3-hexafluoropropane and *n*-C_5_H_12_ at −90 °C. The Cp region (5.7–5.8 ppm has been resolution enhanced to resolve different chemical shifts). Cp resonances from the methylene-bound isomers are marked with *.

When [η^5^-CpOs(CO)_3_]^+^(1) is irradiated at temperatures at or below −90 °C in the presence of pentane, the Cp resonance for (1) at *δ* 5.91 decreases in intensity and a triplet resonance grows in the high field region of the ^1^H NMR spectrum (*δ* −2.34) (Fig. 5). This signal is the only resonance visible in the high field region of the ^1^H NMR spectrum during photolysis.

The triplet splitting pattern of the resonance at *δ* −2.34 is indicative of a bound –CH_3_ with an adjacent –CH_2_– group. The resonance has a splitting ^3^*J*_H–H_ = 6.6 Hz and is very similar to the resonances which have previously been described for methyl, –CH_3_, -bound *n*-pentane σ-complexes.^14,33,57–59^ The resonance at *δ* −2.34 grows in concomitantly with a resonance at *δ* 5.73 in Cp region of the ^1^H NMR spectrum. These resonances reach a maximum after ∼10 minutes of photolysis and are ascribed to the methyl (C1)-bound *n*-pentane σ-complex, [η^5^-CpOs(CO)_2_(*n*-C_5_H_12_)]^+^ (4a) (Fig. 5, see also Fig. S4 in the ESI†).

**Fig. 6:**
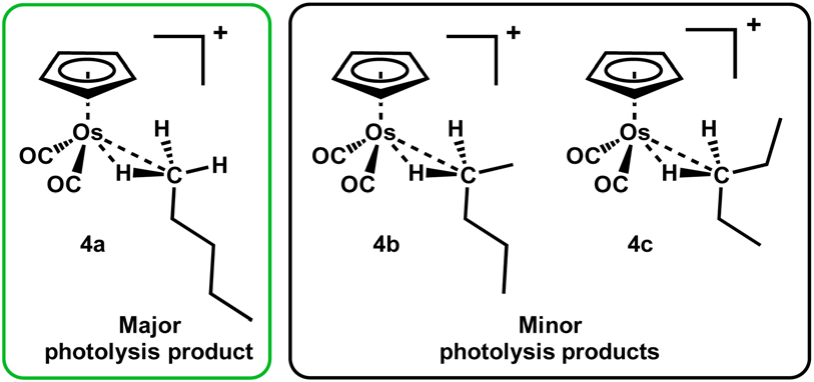
The methyl-bound *n*-pentane is the major product during irradiation of [η^5^-CpOs(CO)_3_]^+^(1) in a solution of 1,1,1,3,3,3-hexafluoropropane saturated with *n*-C_5_H_12_ at −90 °C.

Only very small signals from the cyclopentadienyl protons of the methylene-bound isomers (marked with an asterisk in Fig. 5) are observed in the ^1^H NMR spectrum during irradiation indicating that these species, 4b and 4c, are present but only in small concentrations (Fig. 6).

When irradiation is turned off, the system equilibrates and the resonance at *δ* −2.34 in the ^1^H NMR spectrum rapidly decreases in intensity and two broad resonances at *δ* −4.10 and *δ* −4.42 grow into the high field region of the ^1^H NMR spectrum (total yield of all isomers of *n*-pentane isomer *ca.* 10%). We attribute the two new high-field resonances to the metal-bound C–H of the two methylene-, –CH_2_–, bound isomers of the *n*-pentane complex, [η^5^-CpOs(CO)_2_(*n*-C_5_H_12_)]^+^ (4b and 4c). The Cp resonance of 4a at *δ* 5.73 also decreases in intensity rapidly and a resonance at *δ* 5.74 grows in its place. We attribute the Cp resonance at *δ* 5.74 to an overlapped signal from the two methylene-, –CH_2_–, bound isomers of the *n*-pentane complex, [η^5^-CpOs(CO)_2_(*n*-C_5_H_12_)]^+^ (4b and 4c). When the sample is exposed to UV light for a second time, the resonance at *δ* 5.74 decreases in intensity and the resonance at *δ* 5.73 and the triplet resonance at *δ* −2.34 are restored to their maximum intensities. This photo-driven isomerization is reversible and can be cycled, without any appreciable loss of the total quantity of complex 4. Monitoring the cyclopentadienyl resonances at *δ* 5.73 and *δ* 5.74 immediately after switching off the lamp at −96 °C (ESI Fig. S5†) indicates that the system effectively equilibrates completely within ∼200 s of the irradiation being turned off and 4a has an effective half-life of approximately 40 s to decay to its equilibrium concentration. The equilibration indicates that interconversion of the different isomers 4a–c is occurring at −96 °C, implying that there is facile swapping of which carbon is bound to the metal centre.

The reversibility observed here is indicative of a system where the methyl-bound isomer 4a is the major product formed during photolysis and the methylene-bound products 4b and 4c are thermodynamic products, which form preferentially when the system is allowed to equilibrate. We considered two possible mechanisms to explain the preference for the formation of 4a during photolysis: (i) it is assumed that irradiation of either the starting material [η^5^-CpOs(CO)_3_]^+^(1) or any of the alkane complexes [η^5^-CpOs(CO)_2_(*n*-C_5_H_12_)]^+^ (4a–c) will lead to the formation of the unsaturated fragment [η^5^-CpOs(CO)_2_]^+^. If this fragment kinetically prefers to bind the pentane at C1, this results in the preference for binding at this site. (ii) The methylene-bound isomers 4b and 4c are substantially more readily photolyzed than methyl-bound isomer 4a. This would mean that isomer 4b and 4c are being converted to [η^5^-CpOs(CO)_2_]^+^ at a faster rate during irradiation, resulting a net selection of the less photo-labile isomer 4a. While we would not have predicted a significant difference in the photo-lability of the three different pentane complex isomers 4a–c, it is not possible to easily discriminate between the two mechanisms at this stage.

**Fig. 7:**
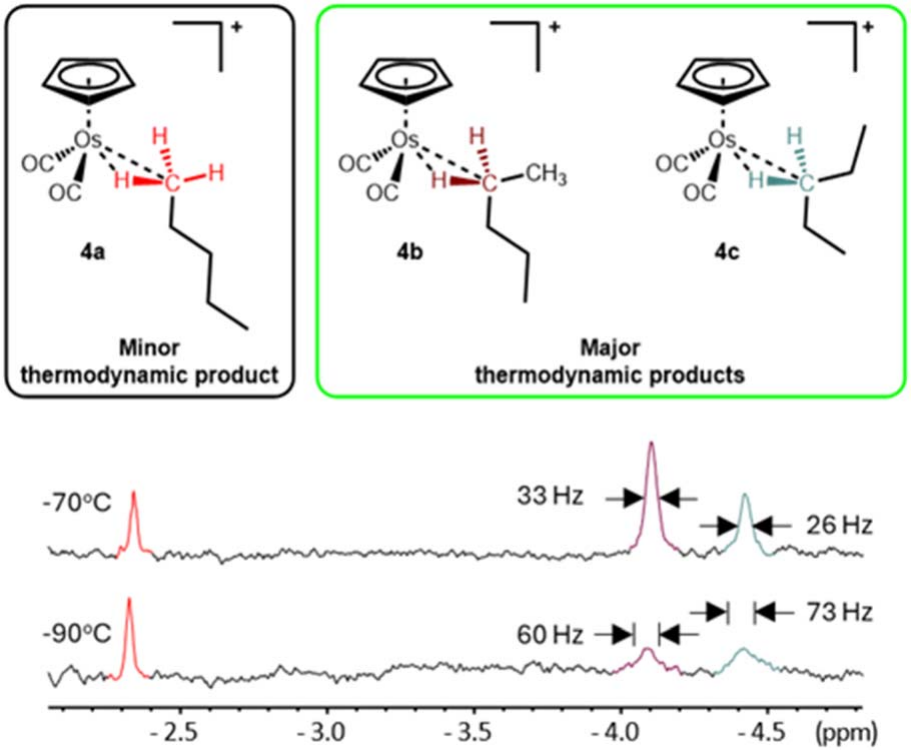
The high field region of the 700 MHz ^1^H NMR spectra of the isomers of the cationic *n*-pentane σ-complex, [η^5^-CpOs(CO)_2_(*n*-C_5_H_12_)]^+^, collected at −90 °C (bottom) and −70 °C (top).

While separate Cp resonances for 4b and 4c were not distinguished, the metal-bound methylene, –CH_2_–, groups have quite distinct chemical shifts in the high field region of the ^1^H NMR spectrum. Once the irradiation was switched off, two broadened resonances at *δ* −4.10 (Δ*ν*_1/2_ = ∼60 Hz) and *δ* −4.42 (Δ*ν*_1/2_ = ∼73 Hz) grow into the high field region of the ^1^H NMR spectrum. At −90 °C, the two high field resonances are difficult to distinguish above the baseline however, at −70 °C, the resonances sharpen, becoming more intense and more visible (Fig. 7).

The broadness of the high field resonances of the coordinated pentane at lower temperatures probably arises from slowing an exchange process to the point where a decoalescence of the NMR signal is about to occur. Three possible exchange process are: (i) the exchange of the geminal –CH_2_– protons of the methylene (one coordinated and one non-coordinated) to the metal centre but given the calculated low energy barrier for this process in the corresponding ethane complex, this process is probably too fast to contribute to the observed broadening; (ii) slowing the exchange rate between different conformations that the pentane could adopt while coordinated to the metal centre (Fig. 9); and (iii) the exchange of the complex between different states of ion pairing or aggregation. The melting point of the solvent (−93 °C for neat HFP) precluded observation of the complexes 4a–c at lower temperatures where the exchange processes may have been frozen out.

At −70 °C, the broad resonances at *δ* −4.10 (Δ*ν*_1/2_ = ∼33 Hz) and *δ* −4.42 (Δ*ν*_1/2_ = ∼46 Hz) appear in roughly a 2 : 1 ratio and, based on the assumption that the two methylene groups will have similar binding affinity for the metal centre and based on data collected for the analogous [η^5^-CpRe(CO)_2_(*n*-C_5_H_12_)] complex,^27,57–59^ we ascribe the resonances at *δ* −4.10 and *δ* −4.42 as the C2-bound and C3-bound isomers 4b and 4c respectively. The high field resonances at *δ* −2.34, *δ* −4.10 and *δ* −4.42 and the Cp resonances at *δ* 5.73 and *δ* 5.74 can all be observed at temperatures as high as −45 °C for tens of minutes before they disappear into the baseline noise. The effective half-lives for 4a–c were measured to be approximately 57 minutes at −50 °C (ESI Graph S3 and Table S3†).

The process of isomerization, to form the equilibrium mixture of 4a, 4b and 4c was not explored in detail during this investigation. The process could involve a sequence of shifts (1,2), (1,3) or (1,4) shifts along the *n*-pentane chain (“chain walking”) as has been previously suggested for the isoelectronic rhenium complexes, [η^5^-CpRe(CO)_2_(*n*-C_5_H_12_)] and [η^6^-(HEB)Re(CO)_2_(c-C_5_H_10_)]^+^. At equilibrium, the integral ratio of the *n*-pentane σ-complexes, 4a, 4b and 4c, is approximately 3.0 : 11.2 : 6.4 respectively and this translates to a molar ratio of 1 : 5.6 : 3.2. These indicate a slight binding preference for the 4c isomer as there is only one binding sites in 4c compared to two binding sites in 4a and 4b.

As with the investigations into both the previously reported osmium methane σ-complex and the ethane σ-complex (1) discussed above, we have not observed any hydride containing species that could result from oxidative cleavage of the coordinated C–H bond in any of the *n*-pentane σ-complexes.

### Coordination of cyclopentane, [η^5^-CpOs(CO)_2_(c-C_5_H_10_)]^+^ (5)

Cyclopentane complexes are amongst the most widely reported metal-alkane σ-complexes.^11,14,30–32,34,48^ Previous investigations into this class of σ-complex have shown that cationic metal complexes and heavier transition metals tend to form more stable cyclopentane σ-complexes.

**Fig. 8:**
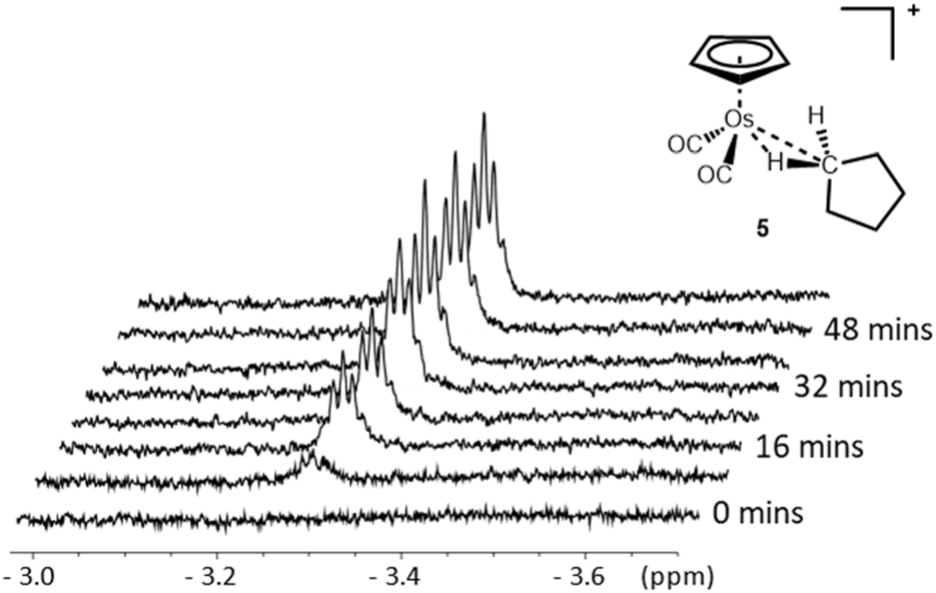
The high-field resonance in the 700 MHz ^1^H NMR spectrum of [η^5^-CpOs(CO)_2_(c-C_5_H_10_)]^+^(5) which grows in during irradiation of [η^5^-CpOs(CO)_3_)]^+^(1) in a solution of 1,1,1,3,3,3-hexafluoropropane saturated with c-C_5_H_10_ at −90 °C.

When [η^5^-CpOs(CO)_3_]^+^(1) is irradiated in a mixture of HFP (∼700 μL) and cyclopentane (∼15 μL), a new resonance grows in the previously empty high-field region of the ^1^H NMR spectrum (Fig. 8) (yield *ca.* 20%). The new resonance appears as a proton-coupled 5-line multiplet at *δ* −3.26 and this signal is ascribed to [η^5^-CpOs(CO)_2_(c-C_5_H_10_)]^+^(5). The high field resonance with the multiplicity shown in Fig. 8 is almost identical to that observed for the previously reported for rhenium, tungsten and manganese complexes with a metal-bound cyclopentane.^11,14,34^

Concomitant with the growth of the resonance in the high field region of the ^1^H NMR spectrum, a number of other resonances grow in with chemical shifts >0. A singlet grows in the cyclopentadienyl region (*δ* 5.60) and two, second-order multiplets grow in the alkyl region (*δ* 1.73 and *δ* 1.68) of the ^1^H NMR spectrum. The resonances at *δ* 1.73 and *δ* 1.68 are assigned to the protons of the non-coordinated methylene groups of the of the bound cyclopentane. The assignment of all the resonances of the bound cyclopentane ring were confirmed using a ^1^H–^1^H TOCSY experiment (ESI Fig. S7†).

Isotopic labelling of the cyclopentane (^13^C-1, >20%) and proton-carbon single bond correlation NMR experiments (^1^H–^13^C HSQC) correlate the ^1^H resonances of the bound cyclopentane to their ^13^C resonances. The high-field resonance in the ^1^H NMR spectrum correlates to a high field resonance in the ^13^C NMR spectrum at *δ* −2.71 (ESI Fig. S6†) and the resonances in the alkyl region at *δ* 1.73 and *δ* 1.68 were correlated to –CH_2_–^13^C resonances at *δ* 24.6 and *δ* 31.1 respectively. The one-bond C–H coupling in the bound methylene group (^1^*J*_C–H_ = 110.9 ± 1.5 Hz) is significantly reduced compared to that of “free” cyclopentane (^1^*J*_C–H_ = 129 Hz) and is smaller than that reported for the analogous neutral and cationic rhenium complexes, [η^6^-(HEB)Re(CO)_2_(c-C_5_H_10_)]^+^, (^1^*J*_C–H_ = 112.7 ± 2.5 Hz).^11,14^ Note the observed ^1^*J*_C–H_ coupling is the average of two different coupling constants, as one hydrogen in the methylene group interacts directly with the metal centre and the other hydrogen does not; the two hydrogens are rendered equivalent in the NMR spectra due to an exchange process corresponding to the process shown in Scheme 4.

The osmium–cyclopentane complex 5 was observed at temperatures as high as −50 °C in ^1^H NMR spectra with a half-life for disappearance of approximately 13 minutes at −51 °C (ESI Graph S4 and Table S4†) before the resonances ascribed to the complex disappear. This observation temperature is significantly higher that observed for the corresponding neutral rhenium complex, [η^5^-CpRe(CO)_2_(c-C_5_H_10_)], (−80 °C) and is comparable to the more recently reported cationic rhenium complex [η^6^-(HEB)Re(CO)_2_(c-C_5_H_10_)]^+^, where the half-life with respect to decomposition was reported as approximately 88 minutes at −62 °C and NMR spectra of this the complex could be observed at temperature of −47 °C for several minutes.^14^

## Calculated binding energies of different alkanes, isomers and conformers

Table 1 shows the calculated binding energies and binding energies relative the binding of methane of different alkanes to the organometallic fragment [η^5^-CpOs(CO)_2_]^+^.

The electronic-only binding energies of the alkanes increase with size of the alkane, with *n*-pentane and cyclopentane binding 8.3–8.5 kcal mol^−1^ more favourably than methane. We have previously shown that the free energy of binding of HFP in vacuum has been calculated to be 4.7 kcal mol^−1^ less favourable than the binding of methane.^15^

Binding free energies are predictably smaller than the electronic-only energies, mostly on account of the entropy penalty involved in combining two species into one. The relative free energies of binding of the heavier alkanes with respect to methane are slightly less exergonic than the electronic-only energies, in part because there is less of an entropy penalty for binding a highly symmetrical methane molecule in comparison with the higher alkanes.

Considering the *n*-pentane complexes, when the relative energies of pentane binding through C1, C2 and C3 (complexes 4a, 4b and 4c respectively), were calculated in vacuum, 4c was found to be lowest in energy with 4a and 4b, 2.3 and 0.4 kcal mol^−1^ higher in energy respectively. The relative free energies in vacuum would suggest that the amount of 4a observable at equilibrium (in the absence of irradiation) should be very small (<1%) relative to the amount of 4c. However, when solvation energy is included in the model, 4a is only 1.1 kcal mol^−1^ higher in energy than 4b and 4c, which have essentially the same calculated binding energies. The solvent may play a role in allowing the C1-bound isomer 4a to be present in sufficient quantities to be observed at equilibrium, due to a slightly more exergonic solvation energy of the C1-bound isomer 4a.

These calculations agree well with the observed relative ratio of these species and support the slight binding preference for the 4b and 4c isomers relative to 4a at equilibrium.

**Fig. 9:**
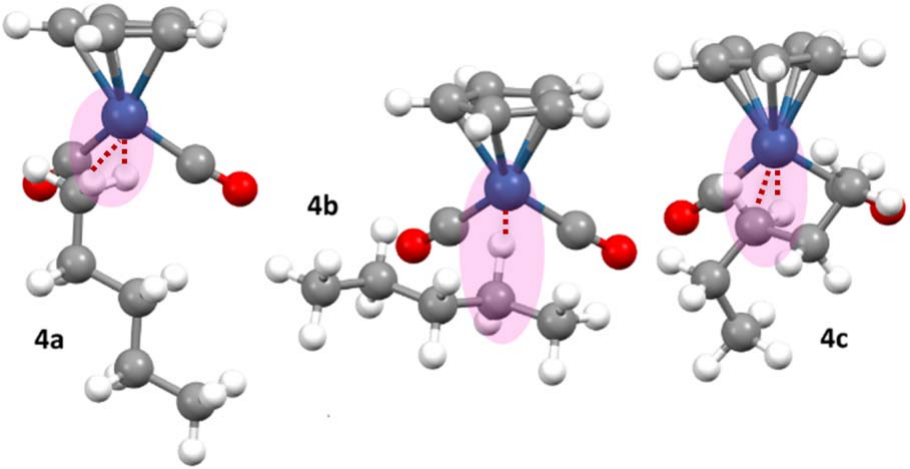
Calculated lowest energy conformations of the three *n*-pentane complexes of the type [η^5^-CpOs(CO)_2_(*n*-C_5_H_12_)]^+^, bound through C1 (4a), C2 (4b) and C3 (4c) of the pentane, located in an SMD model of the 1,1,1,3,3,3-Hexafluoropropane (HFP) solvent.

In arriving at the numbers in Table 1, many different conformations of each pentane isomer were considered. In general, there were several conformations of each isomer found with similar energies, and the same conformation was not the lowest energy conformation for all the calculations in Table 1 – *i.e.* different entries in Table 1 may be different conformations of each *n*-pentane isomer. Multiple conformations of the pentane ligand will be present in solution. Fig. 9 shows the three lowest energy conformations of 4a-c calculated in HFP solvent.

The overall lowest energy *n*-pentane isomer in Fig. 9 is 4c, which notably contains a pentane ligand which is not in the “all *anti*” conformation but rather contains one *gauche* interaction. It is important to consider isomers with *gauche* interactions when modelling these systems, as, even though the [η^5^-CpOs(CO)_2_]^+^ fragment may be considered sterically undemanding, the pentane ligand will in some cases prefer to adopt a conformation with a *gauche* interaction, as the energy penalty associated with the *gauche* interaction is offset by a more favourable interaction between the pentane ligand and the metal centre. Also of note is the different orientation of the C–H bond that is directly interacting with the metal centre in the three different isomers 4a, 4b and 4c. For the dihedral angle defined by the centroid of the Cp-Os-H-C, this value is close to 83° for 4a, 173° for 4b and 44° 4c. This is shown in Fig. 9.

## Conclusion

The work reported here describes the most stable ethane complex reported to date. The cationic osmium-centred σ-ethane complex, [η^5^-CpOs(CO)_2_(C_2_H_6_)]^+^ has an effective half-life of ∼14 hours at −84 °C, making it significantly more stable than the next most stable ethane complex reported, [(PONOP)Rh(C_2_H_6_)]^+^ (*t*_1/2_ = 5.5 h at −132 °C), and substantially more stable than its neutral group 7 neighbour, [η^5^-CpMn(CO)_2_(C_2_H_6_)] (*t*_1/2_ = 360 s at −138 °C). NMR spectroscopy shows that ethane is coordinated to the organometallic fragment, [η^5^-CpOs(CO)_2_]^+^*via* one methyl group and shows no detectable intramolecular exchange between the metal-bound and non-bound methyl groups at −84 °C. There is no evidence for the oxidative cleavage of the coordinated C–H bond in [η^5^-CpOs(CO)_2_(C_2_H_6_)]^+^ to form the corresponding ethyl hydride complex. DFT calculations support the preferred end-on binding of ethane to [η^5^-CpOs(CO)_2_]^+^ and that the barrier to exchange between the bound and unbound methyl groups is much higher than that found in [(PONOP)Rh(C_2_H_6_)]^+^, where exchange of the metal-bound and non-bound methyl groups is rapid even at much lower temperatures.

The σ-pentane and σ-cyclopentane complexes of [η^5^-CpOs(CO)_2_]^+^ are more stable than the ethane complex (as estimated by their respective half-lives in solution and calculated binding energies) and all are more stable than the previously reported methane complex [η^5^-CpOs(CO)_2_(CH_4_)]^+^.^15^ Given the *n*-pentane complex can be observed at temperatures up to −45 °C for tens of minutes, the *n*-pentane complexes are likely the most stable alkane complexes of this type reported to date in solution.

An unexpected preference for the formation of the C1-bound isomer on the *n*-pentane complex, 4a, was observed during continuous photochemical irradiation. When the light source is switched off, an isomerization process occurs. A period of ∼200 s is required to establish a thermal equilibrium in which the metal binds preferentially *via* C3 and C2 (4c and 4b respectively) over binding to C1. This thermal equilibration establishes that migration of the osmium between different carbon sites (C–C swapping) does take place at a relatively slow pace in the pentane complexes at least.

The sequence of alkane binding strength found for [η^5^-CpOs(CO)_2_]^+^ is *n*-pentane ∼ cyclopentane > ethane > methane and this sequence broadly follows the trend in which heavier alkanes bind more strongly, which has emerged from other systems which have been studied experimentally.^8,14,27–35,45–53^

## Data availability

All data needed to evaluate the conclusions in the paper are present in the paper and/or the ESI.†

## Author contributions

Conceptualization: JDW, GEB, LDF. Methodology: JDW, GEB, LDF. Investigation: JDW, GEB, LDF, DM. Visualization: JDW, GEB, LDF. Funding acquisition: GEB, LDF. Project administration: GEB, LDF, JDW. Supervision: GEB, LDF. Writing – original draft: JDW. Writing – review & editing: JDW, GEB, LDF, DM.

## Conflicts of interest

There are no conflicts to declare.

## Supplementary Material

SC-OLF-D5SC00973A-s001
