# Supplementary material for: Coordination of ethane, pentane and cyclopentane to a cationic osmium complex: comparisons in alkane binding
Source: Chem Sci. 2025 Apr 11;16(19):8532–41. doi: 10.1039/d5sc00973a (PMC11997700; doi:10.1039/d5sc00973a)
Supplement: SC-016-D5SC00973A-s001 [file SC-016-D5SC00973A-s001.pdf]

Supplementary Information (SI) for Chemical Science.
This journal is © The Royal Society of Chemistry 2025

Supplementary Information

Coordination of Ethane, Pentane and Cyclopentane to a Cationic Osmium complex: Comparisons in Alkane Binding

James D. Watson, Dejan Mizdrak, Leslie D. Field* and Graham E. Ball*

School of Chemistry, University of New South Wales, Sydney, NSW 2052, Australia.

Correspondence to: L.Field@unsw.edu.au and G.Ball@unsw.edu.au

This PDF file includes:

Methods
Supplementary Text
Figures S1 to S11
Tables S1 to S4
Graphs S1 to S3

Contents

Compounds, compound numbering and abbreviations **4**
Methods and Materials **5**
Supplementary Data **7**
Figure S1. Selected portions of the 700 MHz ^1H NMR spectrum of $[\eta^5\text{-CpOs(CO)}_2(C_2H_6)]^+$ (2) collected in 1,1,1,3,3,3-hexafluoropropane (HFP) saturated with ethane at -90 ℃. 7
Figure S2 An expansion of the high field region of a ^1H-^{13}C HSQC NMR spectrum collected after photolysis of $[\eta^5\text{-CpOs(CO)}_3]^+$ (1), and C_2H_6 in HFP at -90 ℃. 8
Figure S3 An expansion of the mid-field region of a ^1H-^{13}C HSQC NMR spectrum collected after photolysis of $[\eta^5\text{-CpOs(CO)}_3]^+$ (1), and C_2H_6 in HFP at -90 ℃. 9
Half-life of Ethane Complex $[\eta^5\text{-CpOs(CO)}_2(C_2H_6)]^+$ (2) at -84 °C **10**
Graph S1 The natural logarithm of the integral of the Cp resonance ascribed to $[\eta^5\text{-CpOs(CO)}_2(C_2H_6)]^+$, 2, at -84 ℃ as a function of time. 10
Table S1 The effective half-life of $[\eta^5\text{-CpOs(CO)}_2(C_2H_6)]^+$ (2), at -84 ℃. 10
Graph S2 The natural logarithm of the integral of the Cp resonance ascribed to $[\eta^5\text{-CpOs(CO)}_2(C_2H_6)]^+$, 2, at -76 ℃ as a function of time. 11
Table S2 The effective half-life of $[\eta^5\text{-CpOs(CO)}_2(C_2H_6)]^+$ (2), at -76 ℃. 11
Figure S4 Selected regions of the 700 MHz ^1H NMR spectra collected during irradiation of $[\eta^5\text{-CpOs(CO)}_3]^+$ in a mixed solvent solution of 1,1,1,3,3,3-hexafluoropropane (700 μL) and *n*-C_5H_{12} (20 μL)at -90 ℃. * Denotes an unidentified product that grows concomitantly with the alkane complexes during photolysis and persists throughout the experiments. 12
Figure S5 An expansion of the Cp regions of a series of 700 MHz ^1H NMR spectra collected at 40 second intervals immediately after the UV lamp was turned off after photolysis of $[\eta^5\text{-CpOs(CO)}_3]^+$ in a mixed solvent solution of 1,1,1,3,3,3-hexafluoropropane (700 μL) and *n*-C_5H_{12} (20 μL)at -90 ℃. 13
Half-life of n-pentane Complexes $[\eta^5\text{-CpOs(CO)}_2(C_5H_{12})]^+$ (4a-c) at -50 °C **14**
Graph S3 The natural logarithm of the integral of the combination of the resonances ascribed to the Cp of $[\eta^5\text{-CpOs(CO)}_2(C_5H_{12})]^+$ (4b and 4c), at -50 ℃ as a function of time. 14
Table S3 The effective half-life of $[\eta^5\text{-CpOs(CO)}_2(C_5H_{12})]^+$ (4a-c), at -50 ℃. 14
Figure S6 A 700 MHz ^1H-^{13}C HSQC NMR spectrum of $[\eta^5\text{-CpOs(CO)}_2(c\text{-}C_5H_{10})]^+$ (5) with ^{13}C decoupling in F2. 15
Figure S7. (Left) The high field region of a 700 MHz 2D ^1H-^1H TOCSY NMR spectrum of $[\eta^5\text{-CpOs(CO)}_2(c\text{-}C_5H_{10})]^+$ (5), collected at -85 ℃ with a TOCSY mixing time of 20 ms. (Right) The high field region of a 700 MHz 2D ^1H-^1H TOCSY NMR spectrum of $[\eta^5\text{-CpOs(CO)}_2(c\text{-}C_5H_{10})]^+$ (5), collected at -85 ℃ with a TOCSY mixing time of 80 ms. The color of the TOCSY peaks in the spectra illustrate the methylene to which the metal-bound protons correlate in the structural diagrams. 16
Figure S8 Stacked 700 MHz ^1H NMR spectra of the high field region of $[\eta^5\text{-CpOs(CO)}_2(c\text{-}C_5H_{10})]^+$ (5), collected in HFP at different temperatures

(-80 ℃,-70 ℃, -60 ℃,-55 ℃ and -50 ℃). There is negligible change in the intensity of the resonance at δ -3.26 until the sample is warmed above the threshold temperature (-60 ℃). . 17
Half-life of Cyclopentane Complex [η^5-CpOs(CO)$_2$(*c*-C_5H_{10})]$^+$ (5) at -50 °C 18
Graph S4 A plot of the ln(intensity) of the Cp resonance for [η^5-CpOs(CO)$_2$(*c*-C_5H_{10})]$^+$ (5) at -50 ℃ over time. This gives the effective half-life of assuming a first order decay process. . 18
Table S4 Calculation of the effective half-life of [η^5-CpOs(CO)$_2$(*c*-C_5H_{10})]$^+$ (5) at -50 ℃.......... 18
Table S5 summary of the effective half-lives of the osmium alkane complexes 2, 4b, 4c and 5 collected at different temperatures. 19
Computational Methods 20
Figure S9 Calculated lowest energy structure of the osmium-ethane complex, [η^5-CpOs(CO)$_2$(C_2H_6)]$^+$ (2), (MN15/def2-TZVP) 21
Table S5 Summary of selected bond lengths and bond angles of the calculated lowest energy structure of the osmium-ethane complex, [η^5-CpOs(CO)$_2$(C_2H_6)]$^+$ (2), (MN15/def2-TZVP) 21
Figure S10 Free energy profile for the swapping the coordinated C-H bond in [η^5-CpOs(CO)$_2$(C_2H_6)]$^+$, 2, *via* a H-swap (left) or a C-swap (right) mechanism. 2' is a higher energy structure where the bound ethane is binding in a different orientation relative to the metal. (DLPNO-CCSD(T1)/def2-QZVPP//MN15/def2-TZVP) 22
Figure S11 Calculated free energy profile for rotation of the ethane ligand around an Os–H bond in [η^5-CpOs(CO)$_2$(C_2H_6)]$^+$, (2). 2' and 2" are higher energy structures where the bound ethane is binding in a different orientation relative to the metal. (DLPNO-CCSD(T)/def2-QZVPP//MN15/def2-TZVP) 23
Coordinates for Calculated Structures 24
References 30

Compounds, compound numbering and abbreviations

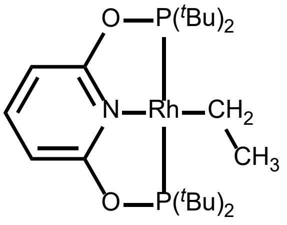

I

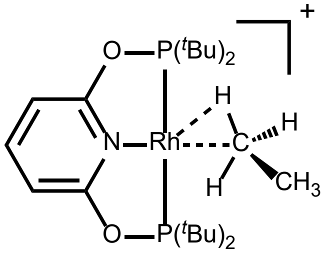

II

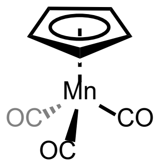

III

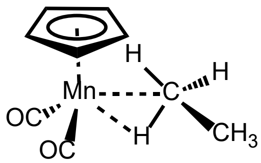

IV

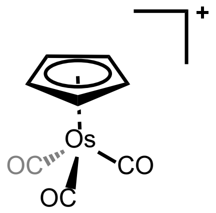

1

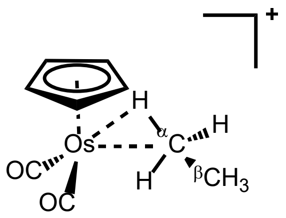

2

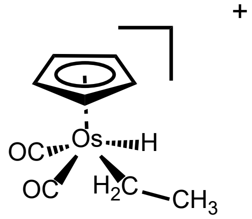

***cis*-3**

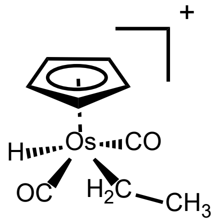

***trans*-3**

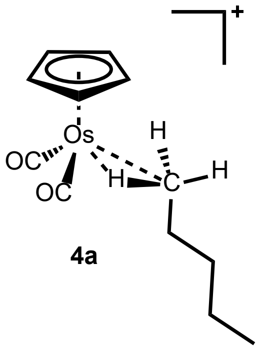

4a

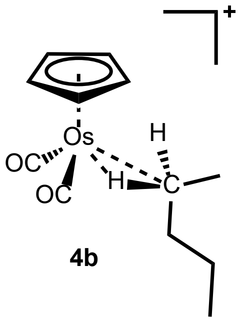

4b

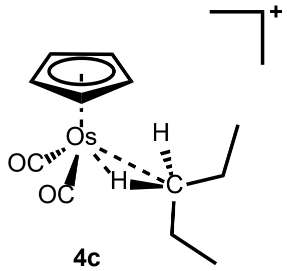

4c

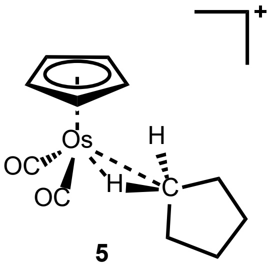

5

$CF_3-CH_2-CF_3$

HFP

1,1,1,3,3,3-hexafluoropropane

Methods and Materials

Acquisition of NMR Data for Alkane σ-Complexes

The NMR experiments that were used to analyze the alkane σ-complexes were performed at low temperatures in *protio*-1,1,1,3,3,3-hexafluoropropane (HFP, $CF_3CH_2CF_3$). HFP does not have any deuterium nuclei and therefore all NMR spectra were collected without a deuterium lock and shimmed by optimizing signals from the ^1H nuclei. To achieve a starting point for shimming on HFP containing samples, the spectrometer was first shimmed (at the experimental temperature, typically around -90 ℃[1]) on a sample of acetone in d_6-acetone (20:80) containing a single drop of CH_2Cl_2 to provide a sharp resonance to use as a reference whilst performing manual shimming. Once the shimming on the acetone:d_6-acetone:DCM sample was acceptable (peak width at half-height of the singlet DCM resonance at δ 5.63 is < 3 Hz), the lock and sweep were turned off, the acetone:d_6-acetone:DCM sample was ejected and the sample for analysis was lowered into the NMR spectrometer using the optical fiber as a tether. Once the sample temperature had settled (~10 minutes) the sample was shimmed manually until the lineshape and peak widths at half height were acceptable ($\Delta\nu_{1/2}$ = < 3 Hz) before the UV lamp was turned on and the sample was irradiated.

Preparation of NMR Samples for Photolysis Experiments

***Precautionary safety note:* 1,1,1,3,3,3-Hexafluoropropane (HFP) boils at ca. 272 K: Samples must be kept at temperatures well below 270 K to mitigate the risk of explosion by the build-up of pressure if $CF_3CH_2CF_3$ is used above its boiling point in a sealed vessel.**

Preparation of Alkane/HFP Solvent Mixtures with Gaseous Alkanes

Mixtures of HFP and ethane were prepared as needed. Solutions of HFP that were saturated with C_2H_6 were produced by filling the headspace of an NMR tube containing HFP at -10 ℃ with C_2H_6 then quickly mixing the NMR tube on a vortex mixer. This process was repeated until there was no longer an increase in the integration of the ^1H signal for the C_2H_6 relative to the ^1H integral for the $CF_3C\mathbf{H_2}CF_3$ resonance. This typically gave a ratio *ca.* 22:1 of $CF_3CH_2CF_3$:C_2H_6.

Preparation of Alkane/HFP Solvent Mixtures with Liquid Alkanes

Mixtures of HFP and liquid alkanes were prepared as needed. *n*-pentane or *c*-pentane (10-15 μL) was first measured into a standard J. Young NMR tube in a nitrogen or argon filled glove box using a micro syringe. The alkane was degassed (using three freeze-pump-thaw cycles). In a separate, pre-dried, standard J. Young NMR tube, HFP (600-700 μL) was vacuum transferred *via* trap-to-trap distillation and then degassed (again using three freeze-pump-thaw cycles). The degassed alkane was transferred *via* trap-to-trap vacuum distillation into the J. Young NMR tube containing the HFP. The solution was allowed to thaw (whilst keeping the

[1] It should be noted that while HFP freezes at ~-91 °C, a combination of supercooling by slowly approaching the freezing point of the solvent and the freezing point depression that arises from the dissolved alkane once facilitated cooling to temperatures as low as -100 °C. However, this was not reliably reproducible as the solution would often freeze if there was even the slighted disturbance of the sample or if there was any precipitation of the complex. For this reason, experiments were conducted at, or around -90 °C.

sample cold) before it was mixed on a vortex mixer. It should be noted that HFP/cyclopentane mixtures containing > ~2 % v/v cyclopentane or > ~5 % v/v pentane will phase separate when the solution is cooled to low temperatures (< -80 °C). This results in droplets of alkane forming and produces a second set of alkane resonances in the ^{1}H and ^{13}C NMR spectra. This does not result in a decrease in the yield of photolysis products but can interfere with the magnetic field homogeneity for the sample and can make shimming more challenging and obscure some resonances in the ^{1}H NMR spectrum.

<u>Sample Preparation for *in situ* Photolysis</u>

NMR samples for photolysis were prepared in J. Young NMR tubes that could be fitted with a modified, bespoke J. Young NMR tube cap (apparatus assembly described previously)[1] with an optical fiber coupling machined from Polyether Ether Ketone (PEEK).

Typically, a modified J. Young NMR tube cap and a pre-dried J. Young NMR tube, were cycled in to an argon- or a nitrogen-filled glove box where 1-2 mg of the desired tricarbonyl precursor complex, $[\eta^5\text{-CpOs(CO)}_3]^+$ was charged into the NMR tube before the tube was sealed using the modified J. Young NMR tube cap. Samples were cycled out of the glove box before being dried *in vacuo* for at least 24 h prior to photolysis in order to remove any volatile contaminants. After the complex had been exposed to a vacuum for an extended period, the desired HFP/alkane solvent mixtures were transferred onto the complex *via* trap-to-trap vacuum distillation. Samples were mixed on a vortex mixer until homogenous at low temperatures below 0 °C.

Samples were connected to the PEEK NMR tube and optical fiber couplers, coupled to an optical fiber connected to a 100 W Hg arc lamp and inserted into a precooled and shimmed NMR spectrometer.

Supplementary Data

Figure S1. Selected portions of the 700 MHz ^1H NMR spectrum of [η^5-CpOs(CO)$_2$(C_2H_6)]$^+$ (2) collected in 1,1,1,3,3,3-hexafluoropropane (HFP) saturated with ethane at -90 °C.

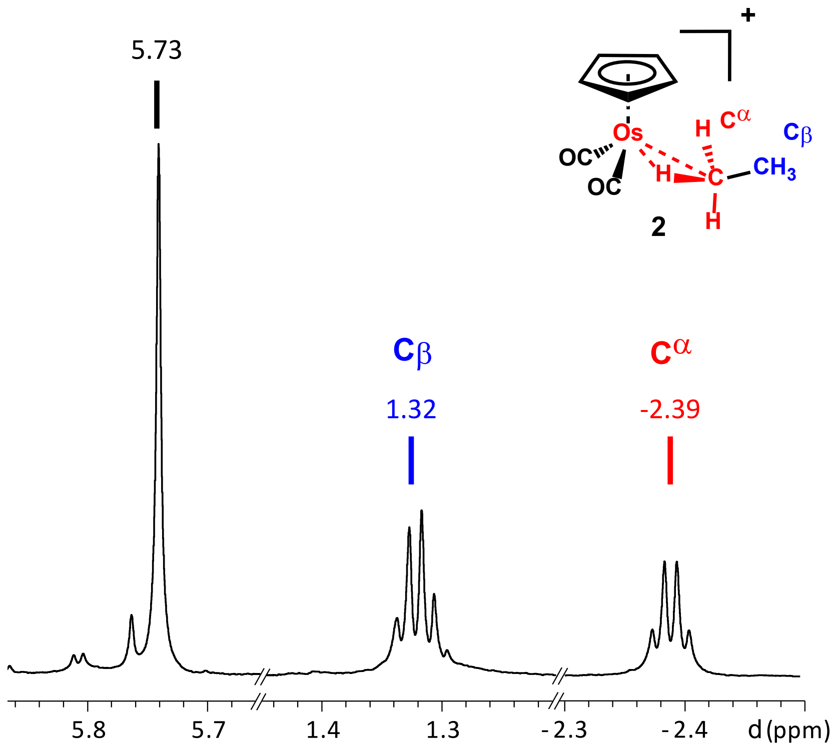

Figure S2 An expansion of the high field region of a ^{1}H-^{13}C HSQC NMR spectrum collected after photolysis of [η[5]-CpOs(CO)$_3$]$^+$ (1), and C_2H_6 in HFP at -90 °C.

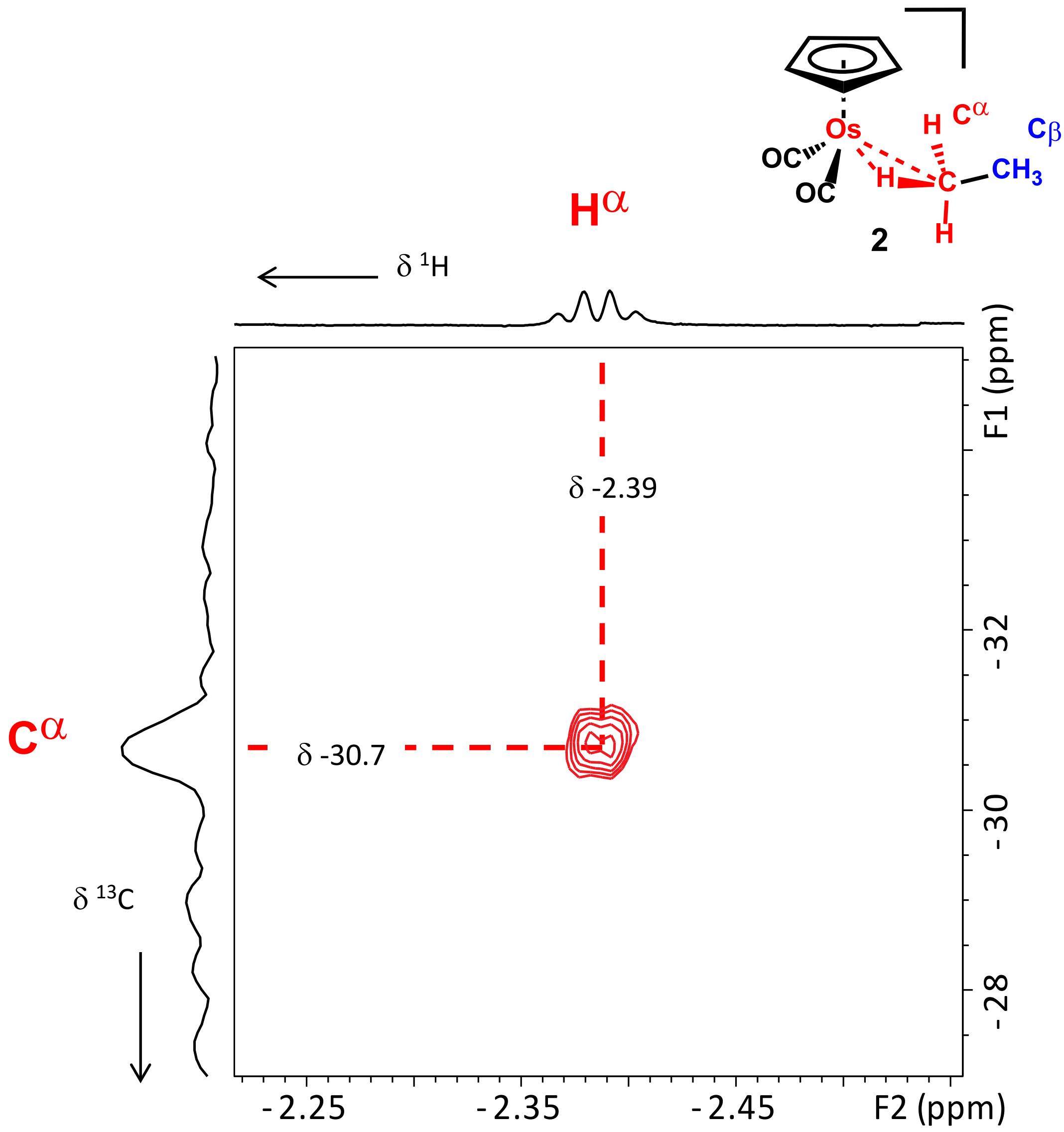

Figure S3 An expansion of the mid-field region of a ^{1}H-^{13}C HSQC NMR spectrum collected after photolysis of [η[5]-CpOs(CO)$_3$]$^+$ (1), and C_2H_6 in HFP at -90 °C.

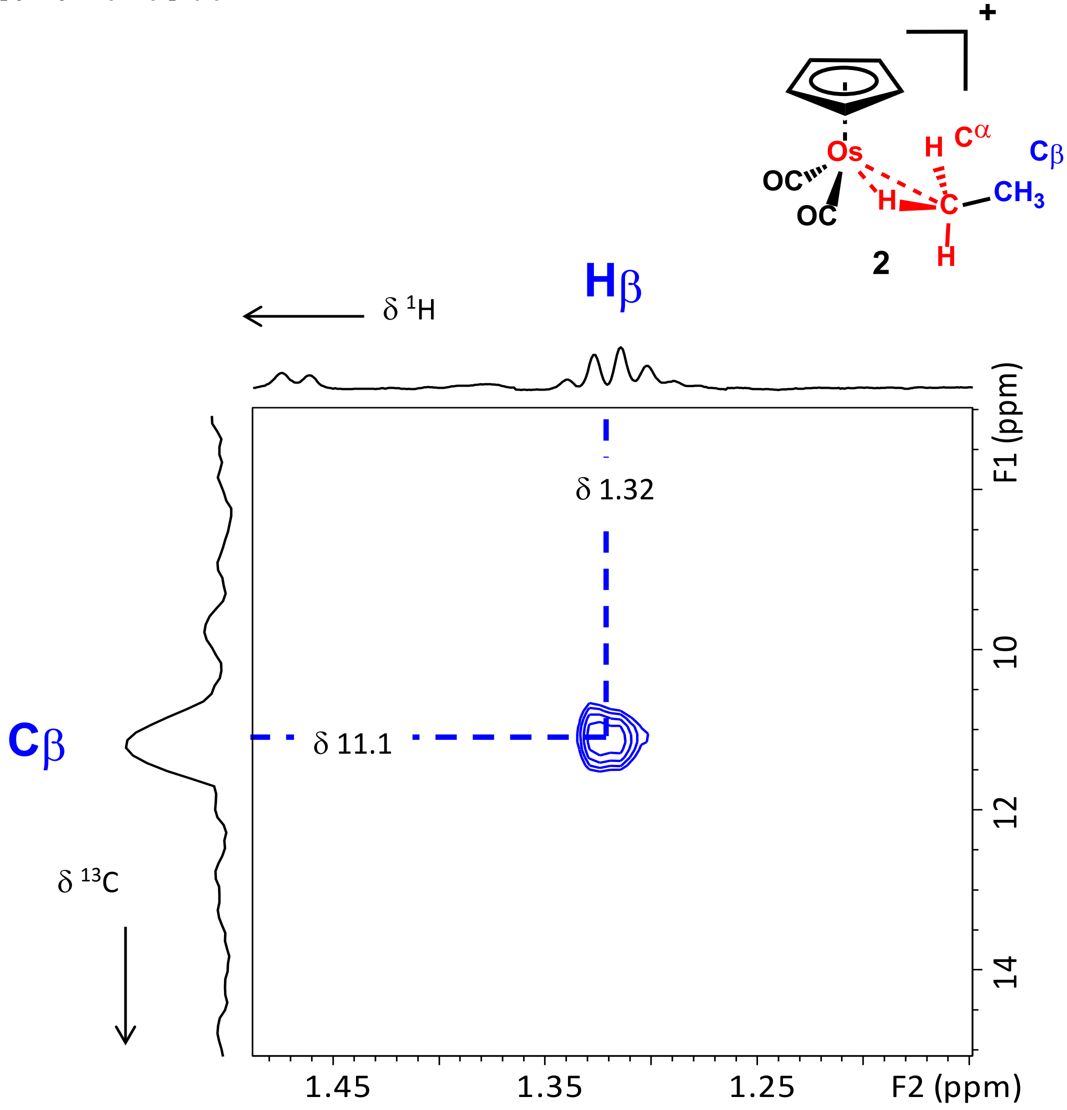

Half-life of Ethane Complex $[\eta^5\text{-CpOs(CO)}_2(C_2H_6)]^+$ (2) at -84 °C

Graph S1 The natural logarithm of the integral of the Cp resonance ascribed to $[\eta^5\text{-CpOs(CO)}_2(C_2H_6)]^+$, 2, at -84 ℃ as a function of time.

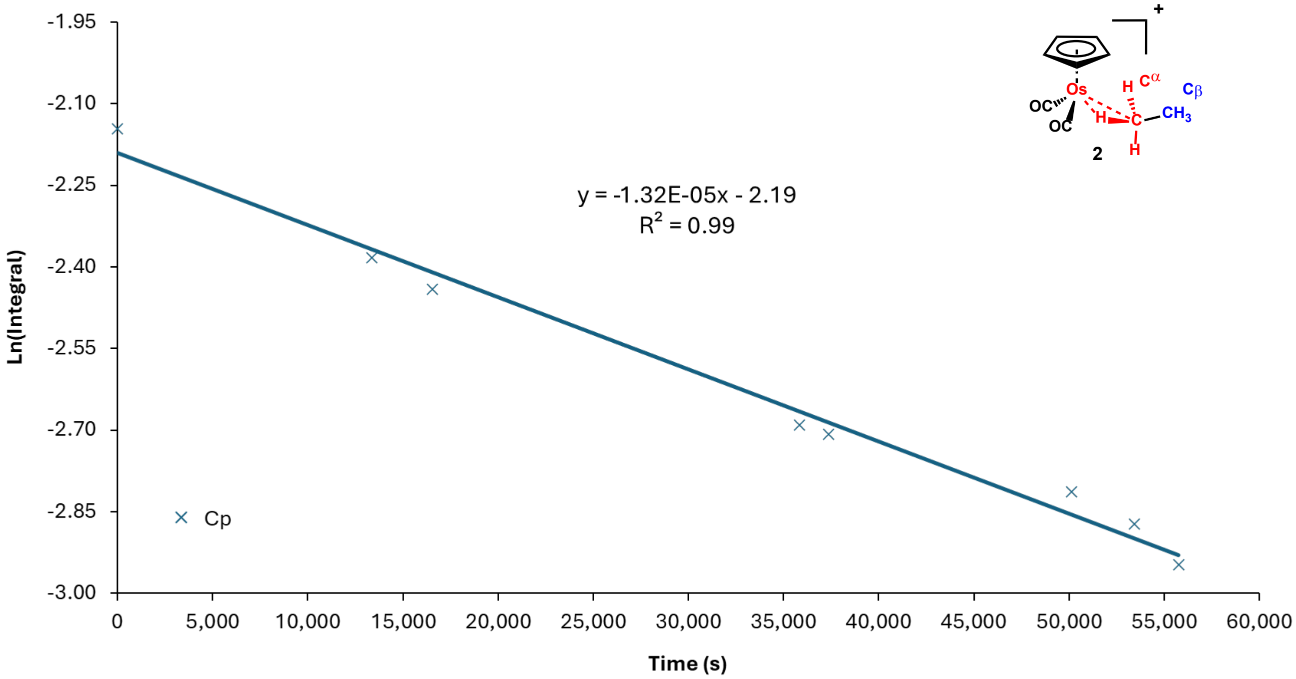

Table S1 The effective half-life of $[\eta^5\text{-CpOs(CO)}_2(C_2H_6)]^+$ (2), at -84 ℃.

$$k = -1.32 \times 10^{-5} \pm 1.53 \times 10^{-6}$$

$$t_{1/2} = \ln(2)/k = 0.693/k$$

Resonance	Approximate effective half-life (Assuming a 1st order decay process)		
	$t_{1/2}$ (hrs)	$t_{1/2}$ high (hrs)	$t_{1/2}$ low (hrs)
Cp	14.5	16.4	13.0

The error in the rate constant is the 95% confidence limit calculated from the line of best fit. $t_{1/2}$ high and $t_{1/2}$ low are the half-lives derived from the upper and lower bounds of the rate constant.

Graph S2 The natural logarithm of the integral of the Cp resonance ascribed to [η^5-CpOs(CO)$_2$(C$_2$H$_6$)]$^+$, 2, at -76 ℃ as a function of time.

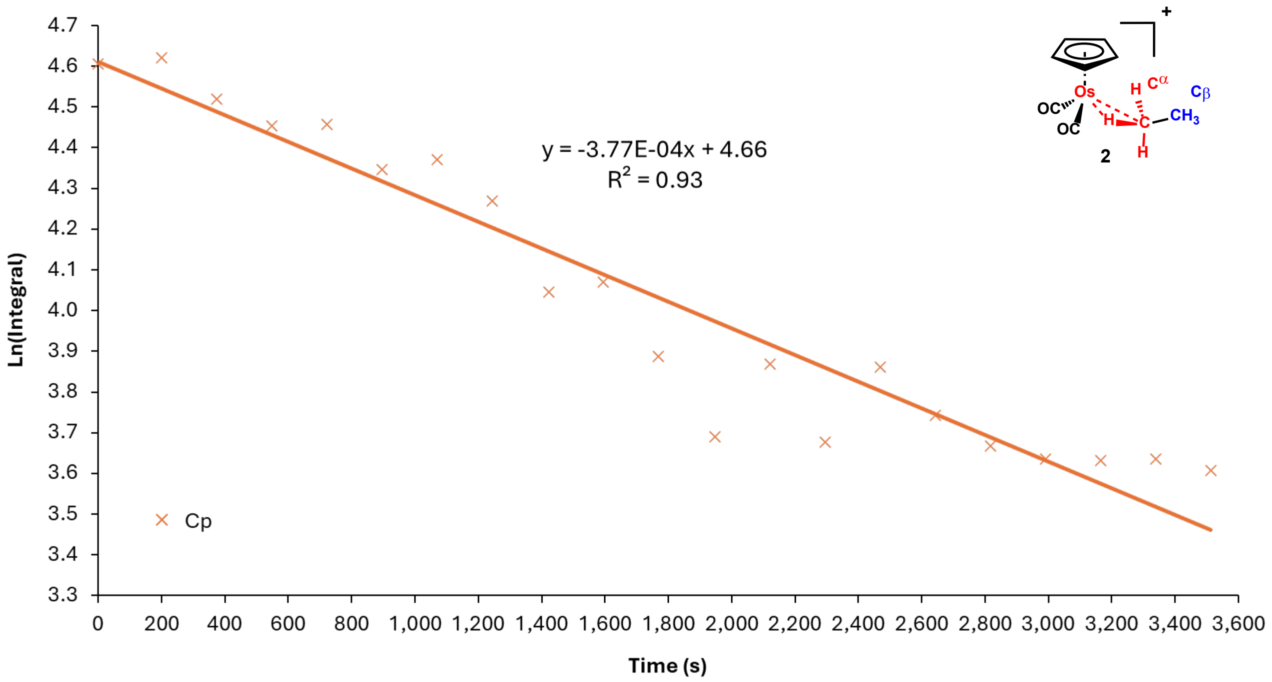

Table S2 The effective half-life of [η^5-CpOs(CO)$_2$(C$_2$H$_6$)]$^+$ (2), at -76 ℃.

$$k = -3.77 \times 10^{-4} \pm 2.78 \times 10^{-5}$$

$$t_{1/2} = \ln(2)/k = 0.693/k$$

Resonance	Approximate effective half-life (Assuming a 1st order decay process)		
	$t_{1/2}$ (hrs)	$t_{1/2}$ high (hrs)	$t_{1/2}$ low (hrs)
Cp	0.59	0.69	0.52

The error in the rate constant is the 95% confidence limit calculated from the line of best fit. $t_{1/2}$ high and $t_{1/2}$ low are the half-lives derived from the upper and lower bounds of the rate constant.

Figure S4 Selected regions of the 700 MHz ^{1}H NMR spectra collected during irradiation of $[\eta^5\text{-CpOs(CO)}_3]^+$ in a mixed solvent solution of 1,1,1,3,3,3-hexafluoropropane (700 µL) and *n*-C_5H_{12} (20 µL)at -90 ℃. * Denotes an unidentified product that grows concomitantly with the alkane complexes during photolysis and persists throughout the experiments.

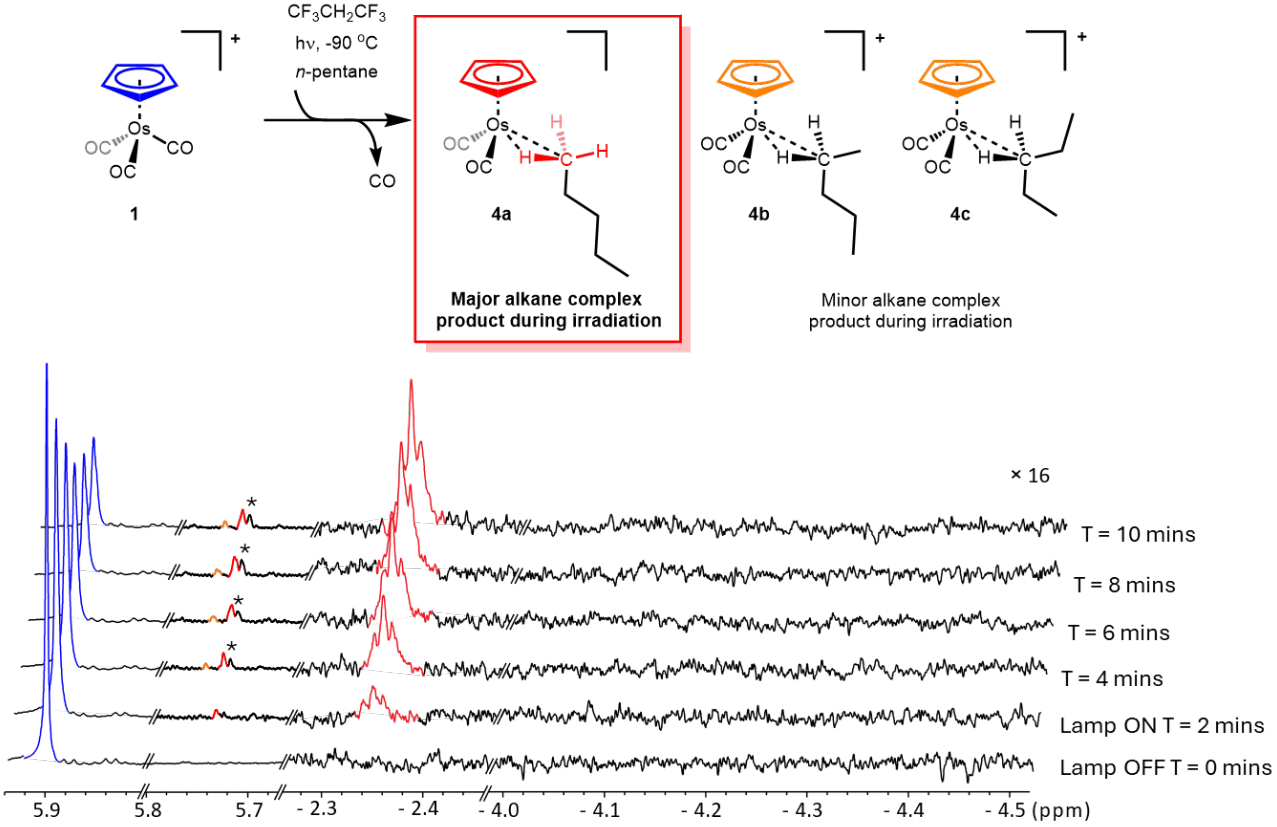

Figure S5 An expansion of the Cp regions of a series of 700 MHz ^{1}H NMR spectra collected at 40 second intervals immediately after the UV lamp was turned off after photolysis of [η^5-CpOs(CO)$_3$]$^+$ in a mixed solvent solution of 1,1,1,3,3,3-hexafluoropropane (700 μL) and *n*-C_5H_{12} (20 μL)at -90 ℃.

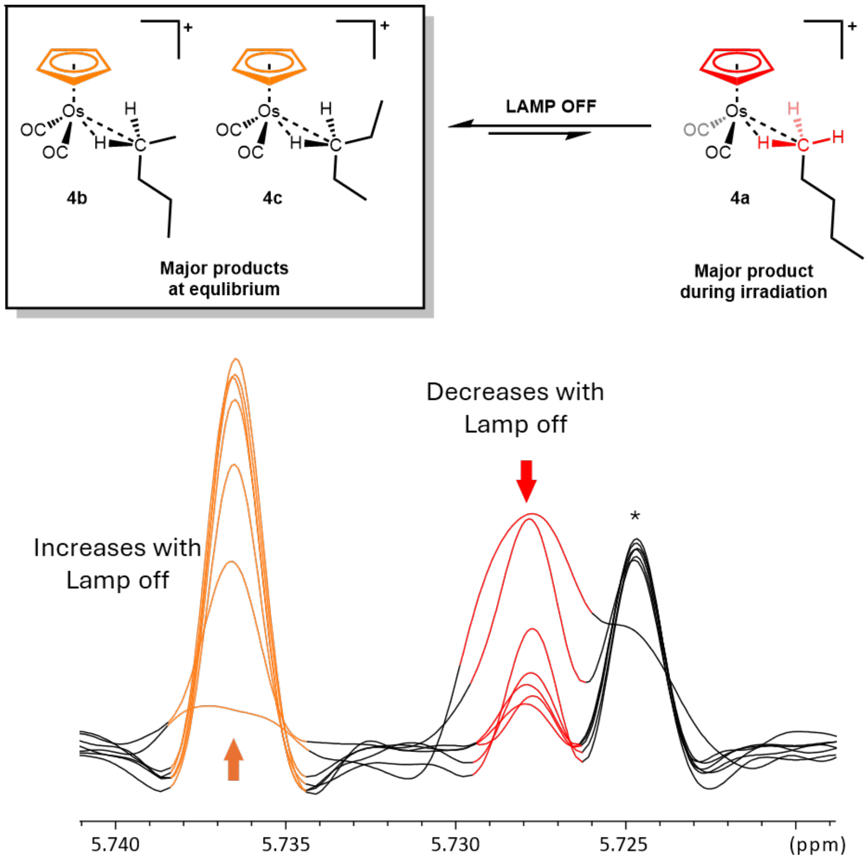

*Denotes an unidentified product that grows concomitantly with the alkane complexes during photolysis and persists throughout the experiments.

Half-life of n-pentane Complexes $[\eta^5\text{-CpOs(CO)}_2(C_5H_{12})]^+$ (4a-c) at -50 °C

Graph S3 The natural logarithm of the integral of the combination of the resonances ascribed to the Cp of $[\eta^5\text{-CpOs(CO)}_2(C_5H_{12})]^+$ (4b and 4c), at -50 ℃ as a function of time.

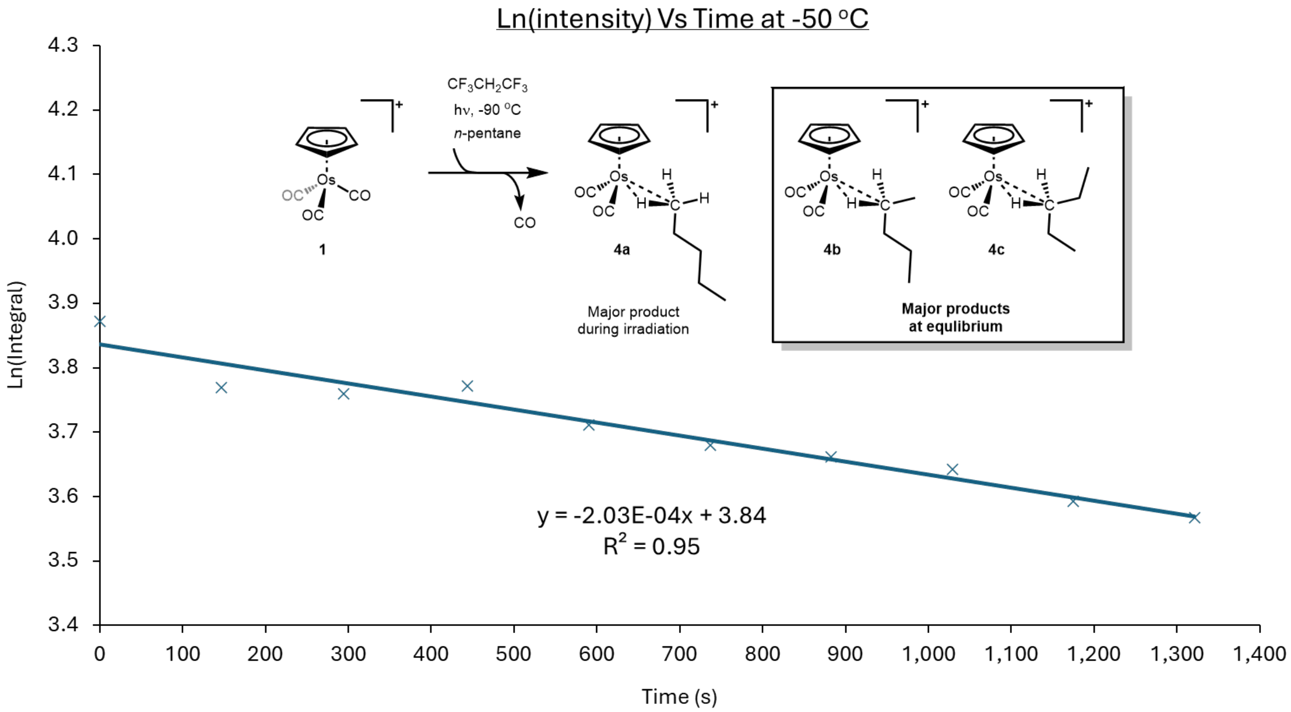

Table S3 The effective half-life of $[\eta^5\text{-CpOs(CO)}_2(C_5H_{12})]^+$ (4a-c), at -50 ℃.

$$k = -2.03 \times 10^{-4} \pm 3.82 \times 10^{-5}$$

$$t_{1/2} = \ln(2)/k = 0.693/k$$

Resonance	Approximate effective half-life (Assuming a 1st order decay process)		
	t1/2 (hrs)	t1/2 high (hrs)	t1/2 low (hrs)
Cp region for 4b and 4c	0.95	1.21	0.77

The error in the rate constant is the 95% confidence limit calculated from the line of best fit. $t_{1/2}$ high and $t_{1/2}$ low are the half-lives derived from the upper and lower bounds of the rate constant.

Figure S6 A 700 MHz ^{1}H-^{13}C HSQC NMR spectrum of [η^{5}-CpOs(CO)$_2$(*c*-C_5H_{10})]$^{+}$ (5) with ^{13}C decoupling in F2.

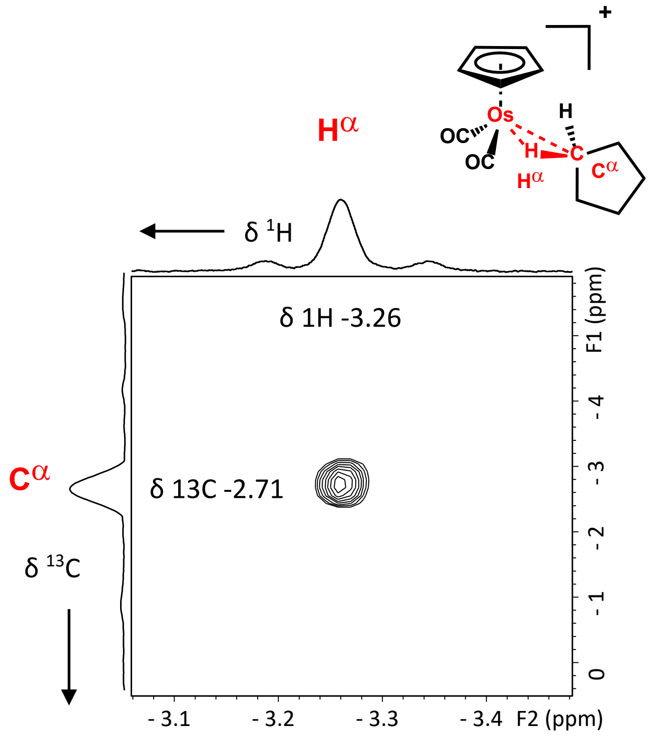

Figure S7. (Left) The high field region of a 700 MHz 2D ^1H-^1H TOCSY NMR spectrum of [η^5-CpOs(CO)$_2$(c-C_5H_{10})]$^+$ (5), collected at -85 ℃ with a TOCSY mixing time of 20 ms. (Right) The high field region of a 700 MHz 2D ^1H-^1H TOCSY NMR spectrum of [η^5-CpOs(CO)$_2$(c-C_5H_{10})]$^+$ (5), collected at -85 ℃ with a TOCSY mixing time of 80 ms. The color of the TOCSY peaks in the spectra illustrate the methylene to which the metal-bound protons correlate in the structural diagrams.

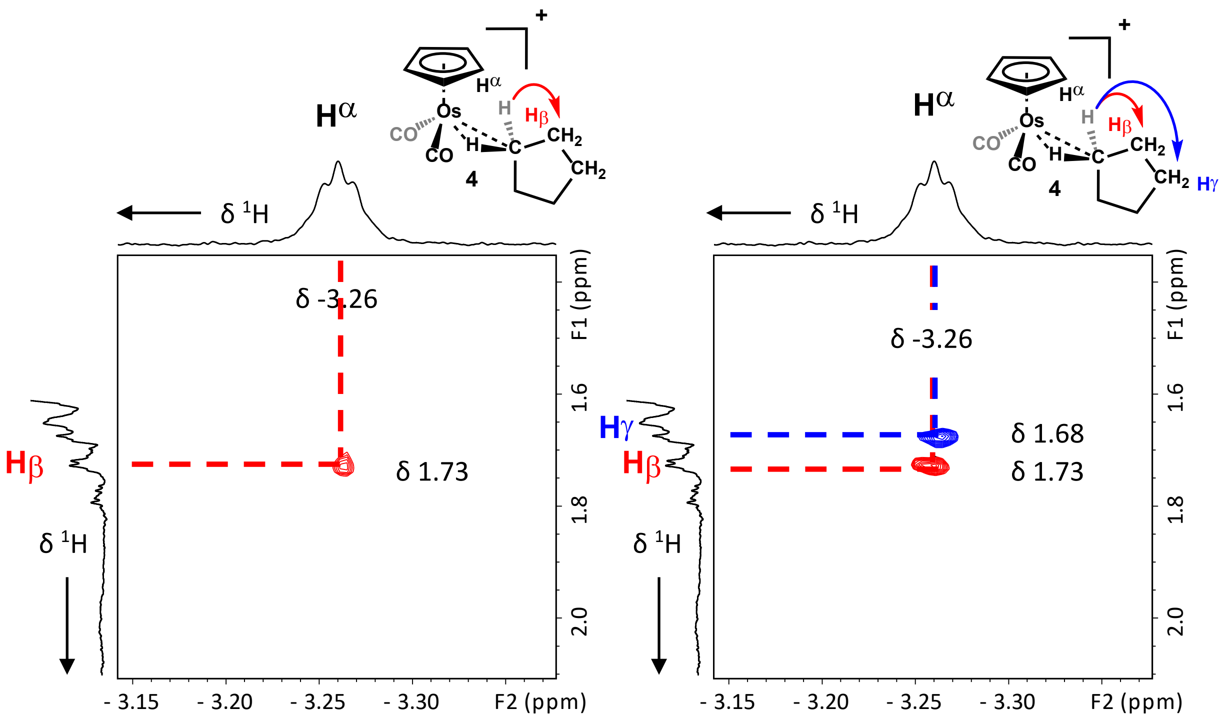

Figure S8 Stacked 700 MHz ^{1}H NMR spectra of the high field region of [η^5-CpOs(CO)$_2$(*c*-C_5H_{10})]$^+$ (5), collected in HFP at different temperatures (-80 ℃,-70 ℃, -60 ℃,-55 ℃ and -50 ℃). There is negligible change in the intensity of the resonance at δ -3.26 until the sample is warmed above the threshold temperature (-60 ℃).

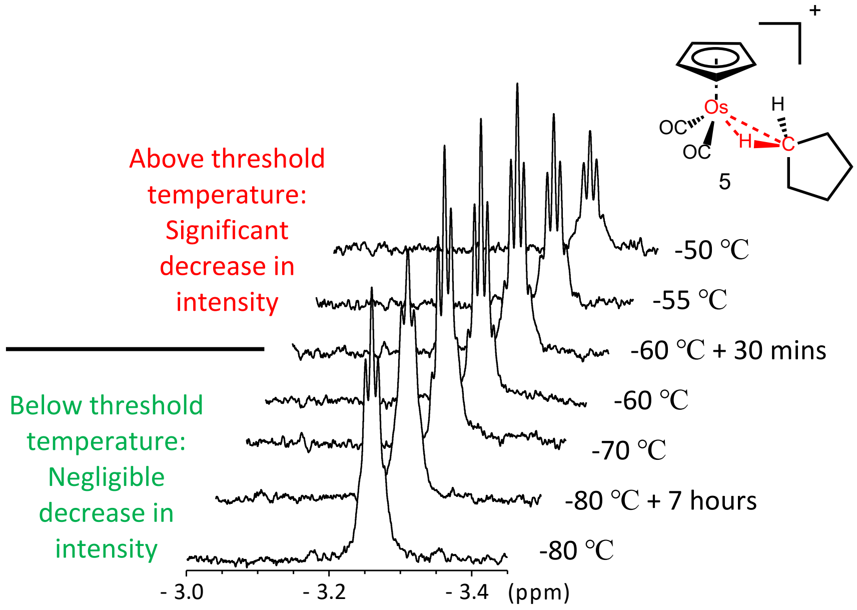

Half-life of Cyclopentane Complex [η^5-CpOs(CO)$_2$(*c*-C_5H_{10})]$^+$ (5) at -50 °C

Graph S4 A plot of the ln(intensity) of the Cp resonance for [η^5-CpOs(CO)$_2$(*c*-C_5H_{10})]$^+$ (5) at -50 °C over time. This gives the effective half-life of assuming a first order decay process.

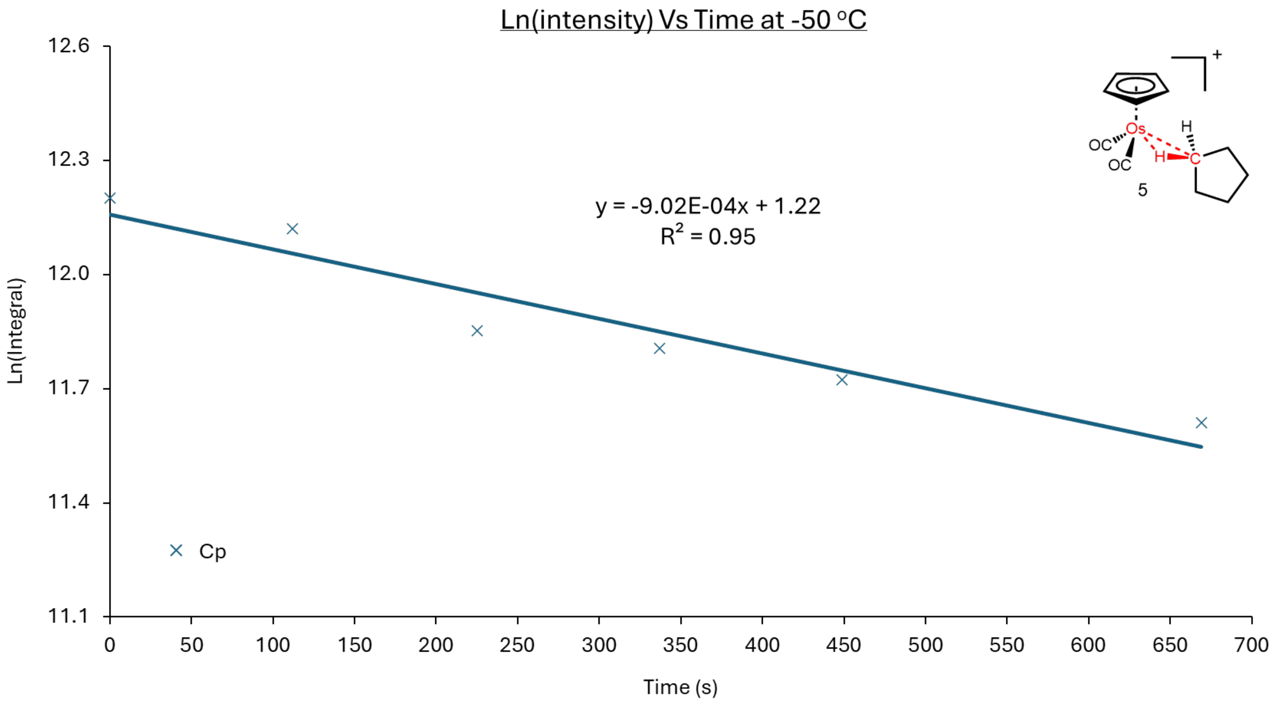

Table S4 Calculation of the effective half-life of [η^5-CpOs(CO)$_2$(*c*-C_5H_{10})]$^+$ (5) at -50 °C.

$$k = -9.02 \times 10^{-4} \pm 2.48 \times 10^{-4}$$

$$t_{1/2} = \ln(2)/k = 0.693/k$$

Resonance	Approximate effective half-life (Assuming a 1st order decay process)		
	$t_{1/2}$ (hrs)	$t_{1/2}$ high (hrs)	$t_{1/2}$ low (hrs)
**Cp**	**0.21**	**0.29**	**0.16**

The error in the rate constant is the 95% confidence limit calculated from the line of best fit. $t_{1/2}$ high and $t_{1/2}$ low are the half-lives derived from the upper and lower bounds of the rate constant.

Table S5 summary of the effective half-lives of the osmium alkane complexes 2, 4b, 4c and 5 collected at different temperatures.

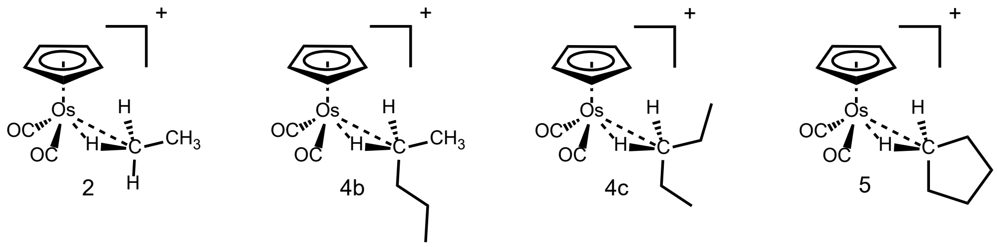

Complex	Temperature (℃)	Approximate effective half-life (Assuming a 1st order decay process)		
		$t_{1/2}$ (hrs)	$t_{1/2}$ high (hrs)	$t_{1/2}$ low (hrs)
**2**	-84	14.5	16.4	13.0
**4b and 4c**	-50	0.95	1.21	0.77
**5**	-50	0.21	0.29	0.16

The error in the rate constant is the 95% confidence limit calculated from the line of best fit. $t_{1/2}$ high and $t_{1/2}$ low are the half-lives derived from the upper and lower bounds of the rate constant.

Computational Methods

Calculations on ethane complexes, isomers and transition structures

Geometry optimizations and frequency calculations were performed using the Gaussian 16 revision C01 software package. (M. J. Frisch. *et al.* (Gaussian, Inc., Wallingford CT, 2013)) employing the MN15 functional[2] and the def2-TZVP basis set with the associated effective core potential (ECP) for the Os atoms.

All minimum energy structures for the complexes analyzed were calculated in vacuum with no symmetry constraints and using the 'ultrafine' grid. The structures were confirmed to be true energy minima by calculating their normal vibrations using the harmonic approximation to confirm that there were no imaginary frequencies. All transition structures have only one imaginary frequency and were determined to be genuine transition structures by running intrinsic reaction coordinate calculations which connected both the staring materials and the products. Frequencies of <50 cm^{-1} were manually replaced with values of 50 cm^{-1} for the purposes of calculating thermodynamic contributions.

The single point energies were calculated using DLPNO-CCSD(T) with def2-QZVPP basis set. DLPNO-CCSD(T) calculations were performed using the ORCA 4.2.1 software using the tightPNO keyword throughout and employing the iterative procedure for improved triples correction (often referred to as DLPNO-CCSD(T1)).[3] Unless otherwise stated, all energies cited in the main text are *free energies* calculated at the DLPNO-CCSD(T)/def2-QZVPP//MN15/def2-TZVP level of theory with vibrational frequencies, enthalpy and entropy calculated using MN15/def2-TZVP.

Calculations on binding energies of different alkanes, isomers and conformers

Calculations on binding energies of different alkanes, isomers and conformers employed the Orca 5.0.4 software. For *n*-pentane complexes, a systematic investigation of different conformations of the pentane complexes was undertaken employing all possible pentane conformations posed in three different ways relative to the $[\eta^5\text{-CpOs(CO)}_2]^+$ fragment. 81 starting geometries of the C1-pentane complex, 29 starting geometries of the C2-pentane complex and 16 starting geometries of the C3-pentane complex were examined. Geometries of each alkane complex/conformation were optimized in vacuum and in SMD HFP solvent using TPSS-D4/def2-TZVP with Orca 5.0.4 software using the keywords tightscf, verytightopt and defgrid3. Low energy conformations of *n*-pentane complexes were identified by comparing single point energies calculated using MN15, wB97M-V and revDSD-PBEP86-D4 methods. Selected low energy geometries (9 C1 pentane complexes, 9 C2 pentane complexes and 5 C3 pentane complexes) were examined at the DLPNO-CCSD(T1)/CBS//TPSS-D4/def2-TZVP level of theory, which includes a complete basis set extrapolation.

Solvation energies of each complex were calculated as the difference of the energy in SMD solvent using the solvated geometry (TPSS-D4/def2-TZVP SMD) and the vacuum energy of the vacuum optimized geometry using the method M05-2X/ 6-31+G(d,p); def2-TZVP for Os atom.

Free energies were calculated with corrections to the electronic energy calculated using TPSS-D4/def2-TZVP at 173 K. Structures displayed below are the conformations with the lowest free energy in solvent found in solution using the DLPNO-CCSD(T1)/CBS//TPSS-D4/def2-TZVP + SMD HFP solvation energy method.

Figure S9 Calculated lowest energy structure of the osmium-ethane complex, [η^5-CpOs(CO)$_2$(C$_2$H$_6$)]$^+$ (2), (MN15/def2-TZVP)

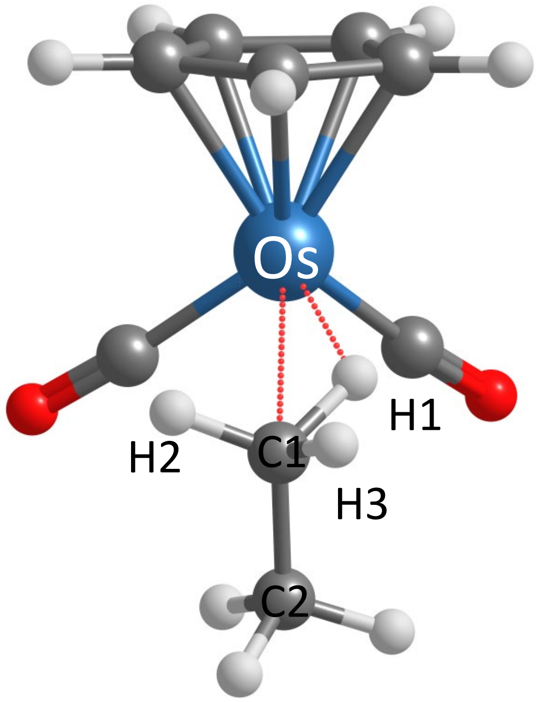

Table S5 Summary of selected bond lengths and bond angles of the calculated lowest energy structure of the osmium-ethane complex, [η^5-CpOs(CO)$_2$(C$_2$H$_6$)]$^+$ (2), (MN15/def2-TZVP)

**Calculated values**			
Bond	Length (Å)	Bond	Angles (°)
Os-C	2.64	Os-H1-C1	121.89
Os-H1	1.84	H1-C1-H2	115.94
Os-H2	2.76	H3-C1-C2	112.78
C1-H1	1.17	H1-C1-H3	95.46
C1-H2	1.09	H1-C1-C2	109.81
C1-H3	1.09	H2-C1-H3	107.35
C1-C2	1.52	H2-C1-C2	114.07

Figure S10 Free energy profile for the swapping the coordinated C-H bond in [η^5-CpOs(CO)$_2$(C$_2$H$_6$)]$^+$, 2, *via* a H-swap (left) or a C-swap (right) mechanism. 2' is a higher energy structure where the bound ethane is binding in a different orientation relative to the metal. (DLPNO-CCSD(T1)/def2-QZVPP//MN15/def2-TZVP)

Barrier to Rotation around the coordinated C-H bond and barrier for C1-C2 C-H Swap

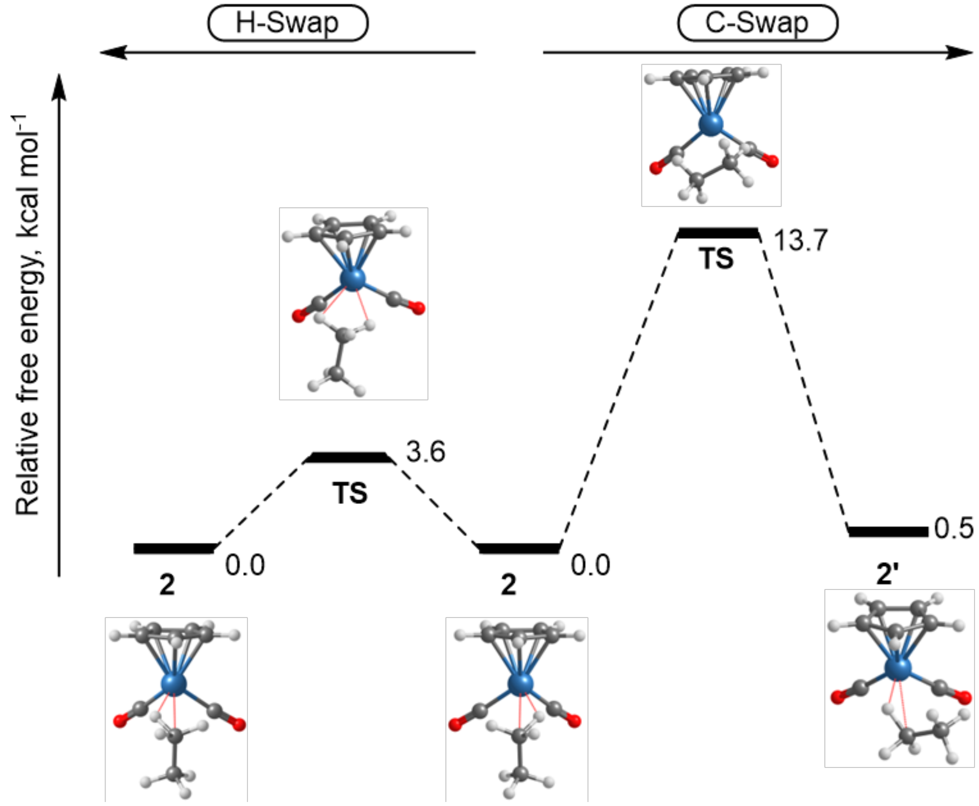

Figure S11 Calculated free energy profile for rotation of the ethane ligand around an Os–H bond in [η[5]-CpOs(CO)$_2$(C$_2$H$_6$)]$^+$, (2). 2' and 2" are higher energy structures where the bound ethane is binding in a different orientation relative to the metal. (DLPNO-CCSD(T)/def2-QZVPP//MN15/def2-TZVP)

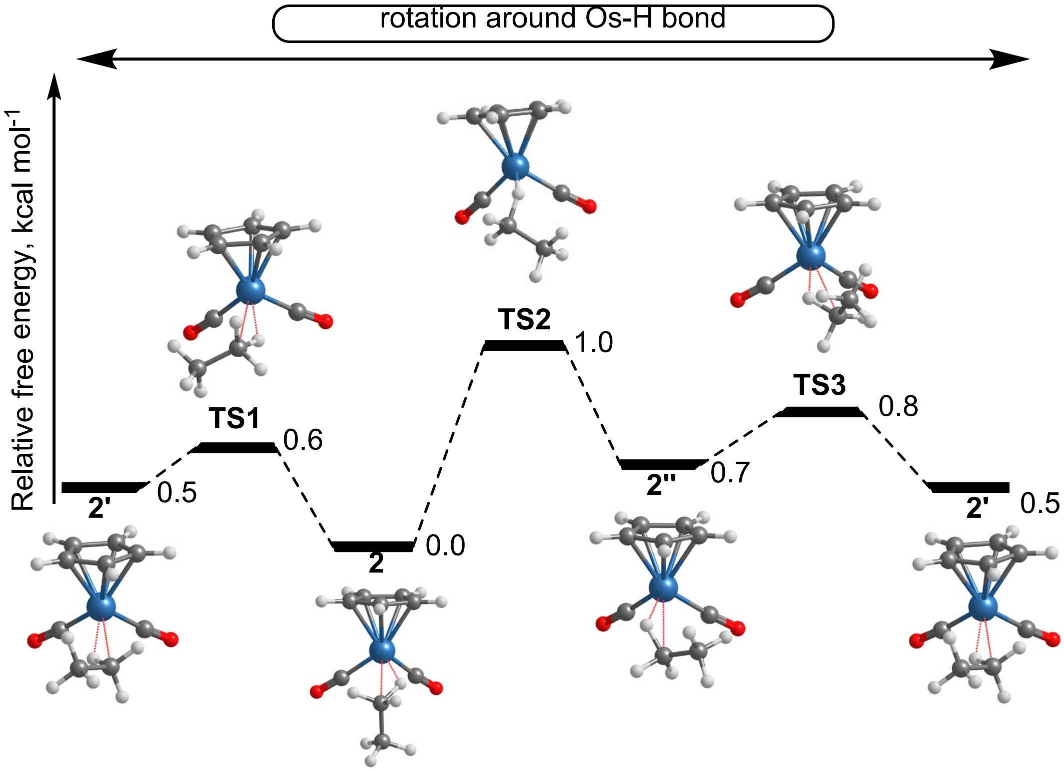

Coordinates for Calculated Structures

Ethane complexes and isomers; MN15 geometries

Osmium ethane complex

[η^5-CpOs(CO)$_2$(C$_2$H$_6$)]$^+$ **2**
Ethane Down
Energy minimum
MN15/def2-TZVP energy:
-590.0641725

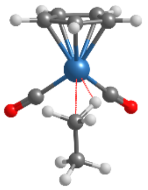

H	1.939862	1.134724	-2.115305
O	-1.222372	2.741369	0.452366
H	1.592613	-1.539438	-2.077104
C	1.920593	0.508928	-1.238333
C	1.777131	-0.915545	-1.215245
C	-0.770198	1.714706	0.277251
C	2.121667	0.931251	0.109365
H	2.279493	1.946054	0.440144
C	1.878325	-1.370959	0.116520
Os	0.115781	0.024380	-0.004532
H	1.831621	-2.396955	0.444486
C	2.069388	-0.216562	0.956349
C	-0.922089	-0.785936	1.412994
O	-1.468066	-1.283154	2.275330
H	2.219929	-0.221279	2.023825
C	-1.866292	-0.923316	-1.475951
H	-1.804278	-1.040634	-2.559325
H	-1.595708	-1.875769	-1.031267
H	-1.061249	-0.082110	-1.409187
C	-3.208146	-0.387178	-1.019839
H	-3.284281	-0.339561	0.066255
H	-3.395400	0.608090	-1.421317
H	-4.000704	-1.044094	-1.377022

Osmium ethane complex

[η^5-CpOs(CO)$_2$(C$_2$H$_6$)]$^+$ **2'**
Ethane Side
Energy minimum
MN15/def2-TZVP energy:
-590.0630184

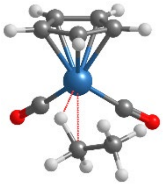

H	-0.212470	-2.566940	1.334293
O	1.685011	0.938029	2.325804
H	-0.503123	-2.550191	-1.331584
C	-0.805667	-1.869966	0.764769
C	-0.991854	-1.877894	-0.641516
C	1.048824	0.657421	1.428176
C	-1.598489	-0.793996	1.290944
H	-1.703880	-0.529228	2.331161
C	-1.883484	-0.832146	-1.007442
Os	-0.096325	0.108114	-0.027622
H	-2.243939	-0.618579	-2.000273
C	-2.262020	-0.157872	0.200396
C	-0.250573	1.925616	-0.661809
O	-0.423973	2.986082	-1.027500
H	-2.949581	0.669249	0.280064
C	2.280649	-0.237875	-1.191598
H	2.521014	-0.082398	-2.245806
H	2.669091	0.615830	-0.646224
H	1.127630	-0.195143	-1.359908
C	2.765532	-1.578306	-0.679215
H	2.596398	-1.691851	0.391149
H	2.276959	-2.403716	-1.196674
H	3.836804	-1.666518	-0.859580

Osmium *cis*-ethyl hydride complex

[η^5-CpOs(CO)$_2$(C$_2$H$_5$)(H)]$^+$ ***cis*-3**
Energy minimum
MN15/def2-TZVP energy:
-590.0453322

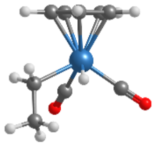

H	1.923635	-1.042231	-2.105379
O	-0.837921	2.943269	-0.630087
H	1.277334	-2.670191	-0.056888
C	1.897937	-0.770171	-1.062202
C	1.563339	-1.633181	0.023551
C	-0.524221	1.878503	-0.404345
C	2.223873	0.503896	-0.520293
H	2.541166	1.366118	-1.085830
C	1.681317	-0.892346	1.229905
Os	0.063101	0.073078	-0.061305
H	1.503183	-1.274219	2.223422
C	2.081858	0.435003	0.902157
C	-1.301197	0.054012	1.306841
O	-2.086820	0.005391	2.122397
H	2.286708	1.228776	1.602330
C	-1.344864	-1.585935	-0.656114
H	-0.821396	-2.090988	-1.464823

H	-1.355411	-2.248922	0.208751
H	-0.602814	0.117366	-1.531105
C	-2.745504	-1.191615	-1.072421
H	-3.282559	-0.653774	-0.290685
H	-2.742979	-0.570220	-1.967622
H	-3.319868	-2.093934	-1.293966

Osmium *trans*-ethyl hydride complex
[η5-CpOs(CO)2(C2H5)(H)]+
***trans*-3**
Energy minimum
MN15/def2-TZVP energy:
-590.0541862

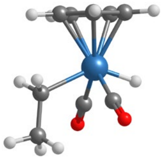

H	-1.245709	0.619474	2.513871
O	1.654387	-2.390187	-0.909156
H	-1.476567	-1.913441	1.624116
C	-1.569049	0.321814	1.528590
C	-1.669856	-1.019547	1.052465
C	1.034518	-1.482827	-0.635692
C	-1.943466	1.201214	0.479982
H	-1.976208	2.277907	0.534970
C	-2.122717	-0.966117	-0.299649
Os	-0.116338	0.002152	-0.190380
H	-2.330916	-1.811577	-0.935447
C	-2.288124	0.409791	-0.650800
C	1.053576	1.470506	-0.688066
O	1.704054	2.352601	-0.969081
H	-2.624966	0.783525	-1.604423
C	1.340384	0.124496	1.525118
H	0.978086	-0.644059	2.206022
H	1.149753	1.097888	1.974383
H	-0.080226	-0.000356	-1.812322
C	2.820141	-0.055478	1.251126
H	3.066709	-1.054433	0.893936
H	3.214028	0.667784	0.536551
H	3.367720	0.091312	2.184666

Transition structure
[η5-CpOs(CO)2(C2H6)]+
Ethane rotation around Os-H bond
MN15/def2-TZVP energy:
-590.0631796

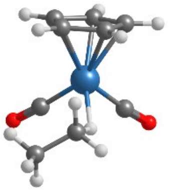

H	0.372205	-2.856497	0.467751
O	0.212346	2.978827	-1.044498
H	1.168409	-1.171058	2.402432
C	0.947435	-1.955060	0.328278
C	1.388198	-1.078499	1.349883
C	0.108591	1.909344	-0.678748
C	1.475077	-1.462995	-0.918255
H	1.322555	-1.908771	-1.889433
C	2.137129	-0.017455	0.748729
Os	0.093647	0.090100	-0.032105
H	2.636766	0.781963	1.270885
C	2.221028	-0.298321	-0.661216
C	-1.126780	0.571634	1.379335
O	-1.798310	0.820974	2.260460
H	2.742844	0.296215	-1.394678
C	-1.919075	-0.929165	-1.393629
H	-2.167984	-0.661799	-2.422009
H	-1.263681	-1.793965	-1.435113
H	-1.396061	0.074497	-1.136803
C	-3.141833	-1.139473	-0.523921
H	-2.876017	-1.512651	0.464529
H	-3.712112	-0.219105	-0.404836
H	-3.794979	-1.874912	-0.993177

Transition structure
[η5-CpOs(CO)2(C2H6)]+
Ethane rotation around Os-H bond
MN15/def2-TZVP energy:
-590.0628548

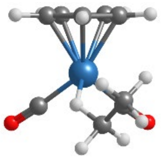

H	0.541137	-2.29072	1.686978
O	0.664715	1.92163	2.313303
H	0.591946	-2.644453	-0.978974
C	-0.178526	-1.909342	0.98103
C	-0.180776	-2.129286	-0.427371
C	0.402167	1.272181	1.419683
C	-1.35489	-1.154404	1.289598
H	-1.667424	-0.835825	2.27179
C	-1.327503	-1.525355	-0.996387
Os	-0.109421	0.095201	-0.022047
H	-1.612783	-1.550595	-2.035485
C	-2.056872	-0.900561	0.073563
C	-0.749553	1.592213	-1.063864
O	-1.203779	2.432068	-1.677147

H	-2.995585	-0.377297	-0.014133
C	2.463539	0.34234	-0.738211
H	2.810806	0.88581	-1.618761
H	2.579512	1.000264	0.115059
H	1.370219	0.203025	-1.114587
C	3.148256	-0.997311	-0.565038
H	2.81738	-1.505794	0.340366
H	2.974274	-1.650012	-1.419902
H	4.223949	-0.842077	-0.484077

Transition structure
$[\eta^5\text{-CpOs(CO)}_2(C_2H_6)]^+$
Ethane rotation around Os-H bond
MN15/def2-TZVP energy:
-590.0625643

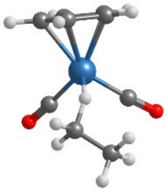

H	1.243973	-2.728906	-0.329394
O	-1.534201	-0.378614	-2.548489
H	1.300171	-1.672926	2.145282
C	1.511398	-1.716168	-0.07672
C	1.583202	-1.162612	1.236582
C	-0.960501	-0.206025	-1.583784
C	1.939765	-0.697348	-0.984749
H	2.023483	-0.793443	-2.055977
C	2.040972	0.173331	1.159513
Os	0.120549	0.040763	-0.004546
H	2.221518	0.834726	1.991219
C	2.252323	0.474011	-0.231249
C	-0.403643	1.897337	0.130026
O	-0.636518	3.005613	0.20867
H	2.632582	1.399273	-0.633766
C	-2.163399	-0.371599	1.440977
H	-2.01582	-0.630168	2.488757
H	-2.282332	0.703431	1.371271
H	-1.174691	-0.794538	0.999089
C	-3.293965	-1.134962	0.779934
H	-3.425439	-0.842969	-0.261371
H	-3.132408	-2.210571	0.822052
H	-4.223916	-0.913696	1.303712

Transition structure
cis-$[\eta^5\text{-CpOs(CO)}_2(C_2H_5)(H)]^+$
***cis*-3**
to $[\eta^5\text{-CpOs(CO)}_2(C_2H_6)]^+$
Interconversion of *cis* ethyl hydride with ethane complex
MN15/def2-TZVP energy:
-590.0439311

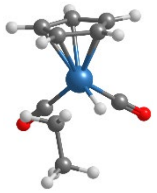

H	2.024303	-0.484141	-2.249628
O	-0.99147	2.877509	-0.604179
H	1.400872	-2.546575	-0.621341
C	1.958177	-0.44733	-1.17418
C	1.64151	-1.542279	-0.308912
C	-0.62444	1.827545	-0.38504
C	2.228499	0.682565	-0.356787
H	2.507037	1.662781	-0.712162
C	1.704085	-1.080544	1.029174
Os	0.071877	0.063329	-0.039483
H	1.529788	-1.676655	1.911489
C	2.057422	0.308112	1.008325
C	-1.208772	-0.037983	1.401126
O	-1.943282	-0.132361	2.260322
H	2.225535	0.936905	1.867472
C	-1.408478	-1.517017	-0.82654
H	-0.977793	-1.965853	-1.719073
H	-1.31587	-2.235338	-0.014272
H	-0.730822	-0.067667	-1.439601
C	-2.844602	-1.092891	-1.045938
H	-3.28489	-0.639549	-0.157952
H	-2.941199	-0.385828	-1.869769
H	-3.441985	-1.973326	-1.290996

Transition structure
trans-$[\eta^5\text{-CpOs(CO)}_2(C_2H_5)(H)]^+$
***trans*-3**
to $[\eta^5\text{-CpOs(CO)}_2(C_2H_6)]^+$
Interconversion of *trans* ethyl hydride with ethane complex
MN15/def2-TZVP energy:
-590.0091164

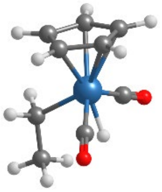

H	-1.14667	0.00052	2.64087
O	0.99004	-2.67391	-1.22887
H	-1.64018	-2.16853	1.13885
C	-1.48624	0.00073	1.61716
C	-1.70962	-1.14294	0.81055

C	0.63917	-1.68448	-0.79963
C	-1.70829	1.14469	0.81057
H	-1.63768	2.17020	1.13888
C	-2.16982	-0.69743	-0.49408
Os	0.00204	-0.00007	-0.09122
H	-2.45926	-1.33703	-1.31299
C	-2.16901	0.69972	-0.49406
C	0.64104	1.68346	-0.80009
O	0.99305	2.67236	-1.22961
H	-2.45770	1.33965	-1.31298
C	1.52622	-0.00038	1.45038
H	1.32781	-0.88022	2.06441
H	1.32809	0.87959	2.06432
H	1.40161	-0.00107	-0.92573
C	2.97032	-0.00061	0.98276
H	3.20499	-0.88399	0.38371
H	3.20534	0.88284	0.38396
H	3.65119	-0.00088	1.83617

Transition structure
$[\eta^5\text{-CpOs(CO)}_2(C_2H_6)]^+$
H-Swap
MN15/def2-TZVP energy:
-590.0585288

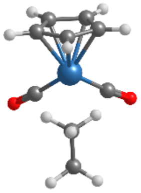

H	1.940345	1.290357	-1.967714
O	-1.133309	2.794067	0.367659
H	1.493737	-1.341268	-2.230527
C	1.913764	0.567962	-1.168456
C	1.720402	-0.832148	-1.304941
C	-0.726224	1.742536	0.238360
C	2.120898	0.830814	0.227993
H	2.328943	1.791206	0.672518
C	1.812426	-1.453849	-0.031108
Os	0.115502	0.008503	0.047650
H	1.750586	-2.510735	0.170176
C	2.059135	-0.414486	0.928102
C	-0.844181	-0.649526	1.596000
O	-1.322778	-1.044654	2.546259
H	2.212242	-0.547186	1.987449
C	-1.818106	-0.767786	-1.486796
H	-1.695636	-1.306241	-2.426353
H	-1.349818	-1.446983	-0.744633
H	-1.271037	0.180655	-1.667252
C	-3.262149	-0.480855	-1.103933
H	-3.322762	0.058525	-0.157633
H	-3.750101	0.125015	-1.865373
H	-3.824257	-1.406909	-0.996993

Transition structure
$[\eta^5\text{-CpOs(CO)}_2(C_2H_6)]^+$
H-Swap
MN15/def2-TZVP energy:
-590.0422538

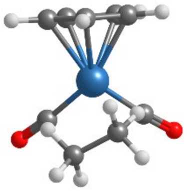

C	1.600632	-1.311793	-0.861796
C	1.308322	-1.743574	0.462481
C	1.623068	-0.714638	1.384718
C	2.101938	0.404188	0.621215
C	2.095411	0.027371	-0.764879
C	-2.651272	-1.08354	0.841853
C	-2.24806	-2.005298	-0.307438
H	1.499877	-1.896202	-1.76165
H	0.859944	-2.692402	0.720861
H	1.538927	-0.770026	2.457735
H	2.458311	1.342935	1.014836
H	2.43194	0.646169	-1.581926
H	-1.816312	-0.898786	1.533832
H	-3.101245	-2.244427	-0.94124
H	-1.827932	-2.941826	0.058823
C	-0.606035	0.928358	-1.626335
C	-0.639277	1.504341	1.104015
O	-0.954004	2.35401	1.785788
O	-0.902324	1.427905	-2.600257
H	-1.499317	-1.555833	-0.981302
H	-3.432873	-1.536206	1.451951
Os	0.069357	0.084037	-0.014018
H	-3.040158	-0.128056	0.486209

Alkane complexes, binding energies; TPSS-D4 geometries

Methane complex
[η^5-CpOs(CO)$_2$(CH$_4$)]$^+$
TPSS-D4/def2-TZVP energy:
-551.5637726

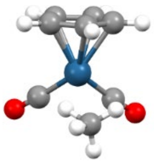

H	1.278276	-0.26784	-2.52581
O	-2.08269	-1.65723	-1.53762
H	1.798643	-2.2509	-0.72812
C	1.497882	-0.13826	-1.47483
C	1.785487	-1.18922	-0.52292
C	-1.36653	-1.03329	-0.89427
C	1.636889	1.119879	-0.79329
H	1.509084	2.095279	-1.244
C	2.060223	-0.56854	0.724621
Os	-0.06142	-0.00199	0.072303
H	2.271284	-1.08908	1.650921
C	1.972192	0.857483	0.578032
C	-1.20207	1.537414	0.009156
O	-1.8138	2.504523	-0.08036
H	2.172263	1.594562	1.342973
C	-1.13864	-1.12369	2.184538
H	-2.21377	-1.19831	2.041119
H	-0.88297	-0.96154	3.235297
H	-0.62142	-2.0058	1.816681
H	-0.81076	-0.08134	1.791556

Ethane complex (2)
[η^5-CpOs(CO)$_2$(C$_2$H$_6$)]$^+$
TPSS-D4/def2-TZVP energy:
-590.9044724

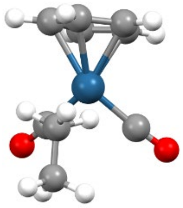

H	1.975465	1.183241	-2.08716
O	-0.98595	2.93483	0.493307
H	1.453455	-1.46775	-2.14576
C	1.941038	0.521105	-1.23351
C	1.702343	-0.89334	-1.26178
C	-0.62852	1.858156	0.315567
C	2.191174	0.87903	0.135888
H	2.422138	1.869651	0.504659
C	1.799425	-1.41498	0.056981
Os	0.115688	0.113707	0.044378
H	1.691578	-2.45128	0.34632
C	2.088993	-0.30698	0.939935
C	-0.90237	-0.57729	1.519141
O	-1.43102	-1.00991	2.442069
H	2.252764	-0.36408	2.00737
C	-1.84729	-0.87937	-1.46662
H	-1.65344	-0.90069	-2.54305
H	-1.47358	-1.80345	-1.02851
H	-1.24513	0.095343	-1.20785
C	-3.30058	-0.59289	-1.11036
H	-3.46237	-0.59709	-0.02985
H	-3.62982	0.368434	-1.51204
H	-3.92636	-1.37737	-1.54866

C1 pentane complex (4a)
[η^5-CpOs(CO)$_2$(C$_5$H$_{12}$)]$^+$
TPSS-D4/def2-TZVP energy:
-708.9188270

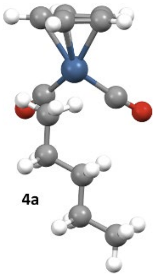

C	2.439972	0.225465	-1.309337
C	2.382997	-1.183429	-1.059617
C	0.231290	-0.549675	2.064325
C	3.029926	-0.165249	0.911138
C	2.748415	-1.440077	0.292067
C	2.848264	0.856014	-0.081451
C	-1.073545	-1.658940	-0.572375
H	3.005808	1.915500	0.070408
H	2.823593	-2.410309	0.763272
H	-0.636372	-0.565241	-0.593031
H	2.254450	0.717897	-2.253451
H	3.360396	-0.006483	1.928575
H	2.074712	-1.933238	-1.777931
H	-0.481856	-2.411671	-0.053587
O	-0.082667	-0.809465	3.138153
Os	0.900434	-0.115059	0.319164
C	-0.031085	1.557189	0.351315
O	-0.502663	2.604579	0.364378
C	-2.485417	-1.503516	-0.015015
H	-2.444334	-1.377833	1.073037
H	-1.044988	-1.860757	-1.647647
C	-3.271380	-0.355579	-0.654072
H	-2.739212	0.592102	-0.480822
H	-3.304996	-0.498262	-1.743113
H	-2.998525	-2.457663	-0.198637
C	-4.699461	-0.234371	-0.108373

H	-4.658062	-0.102560	0.981046
H	-5.232447	-1.177172	-0.287157
C	-5.473187	0.925579	-0.741093
H	-6.486619	0.988365	-0.334441
H	-4.975128	1.883008	-0.549825
H	-5.554597	0.799464	-1.826473

C2 pentane complex (4b)
[η^5-CpOs(CO)$_2$(C$_5$H$_{12}$)]$^+$
TPSS-D4/def2-TZVP energy:
-708.923450

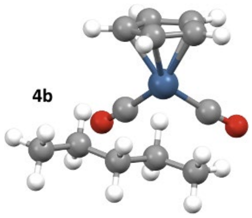

C	-1.741524	0.874362	0.658746
C	-1.513807	-0.505520	0.977273
C	-1.565045	-0.333852	-1.32616
C	-1.415069	-1.257333	-0.22515
C	-1.781587	0.975396	-0.77491
H	-1.952044	1.877798	-1.34676
H	-1.264393	-2.325056	-0.30263
H	-1.900922	1.677637	1.364021
H	-1.565841	-0.585861	-2.37776
H	-1.402013	-0.906598	1.977009
Os	0.252929	0.289294	-0.24174
C	1.481699	-0.635652	-1.38805
O	2.145158	-1.218088	-2.12323
C	1.057247	1.976016	-0.65001
O	1.463300	3.017669	-0.91776
C	2.350396	0.120504	1.65953
H	3.011787	0.166899	0.793761
H	1.213469	0.111636	1.299981
C	2.478715	-1.216909	2.382352
H	3.491932	-1.228735	2.810301
H	1.784039	-1.244225	3.231339
C	2.287602	-2.446112	1.492879
H	3.000057	-2.406482	0.658805
H	1.281776	-2.405820	1.046818
C	2.473440	1.360657	2.534148
H	1.740163	1.352456	3.345426
H	3.473511	1.365035	2.982414
H	2.360449	2.283642	1.960982
C	2.457600	-3.762746	2.257049
H	2.302872	-4.620604	1.596574
H	3.464313	-3.842805	2.679749
H	1.739992	-3.836001	3.081597

C3 pentane complex (4c)
[η^5-CpOs(CO)$_2$(C$_5$H$_{12}$)]$^+$
TPSS-D4/def2-TZVP energy:
-708.9239602

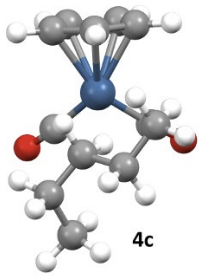

C	1.556824	2.271802	0.770628
C	2.242286	1.429449	1.688072
C	0.622246	2.561546	2.88288
C	1.679004	1.59169	2.995153
C	0.532522	2.972276	1.510797
H	-0.151542	3.710697	1.115579
H	2.017661	1.115442	3.904527
H	1.772710	2.381512	-0.28291
H	0.001802	2.926506	3.690469
H	3.042351	0.748016	1.430295
Os	0.028280	0.823603	1.638291
C	-1.310935	0.269902	2.884697
O	-2.097167	0.019433	3.685092
C	-1.253687	0.875142	0.216855
O	-2.004294	0.995566	-0.64468
C	0.805955	-1.81473	1.388541
H	1.658684	-1.46029	1.970745
H	0.094633	-0.92046	1.071428
C	1.258292	-2.32238	0.016699
H	1.797286	-3.26078	0.207999
H	0.384133	-2.58077	-0.58891
C	2.160023	-1.35137	-0.74118
H	3.082302	-1.1474	-0.18569
H	1.644662	-0.39984	-0.92323
C	-0.072688	-2.75806	2.211377
H	0.513988	-3.67603	2.353929
H	-0.222543	-2.33287	3.209213
C	-1.420525	-3.09435	1.570094
H	-2.004716	-3.73966	2.230998
H	-2.011130	-2.18812	1.387801
H	-1.300412	-3.6167	0.616949
H	2.443495	-1.76357	-1.7131

cyclopentane complex (5)
[η^5-CpOs(CO)$_2$(C$_5$H$_{10}$)]$^+$
TPSS-D4/def2-TZVP energy:
-707.7107852

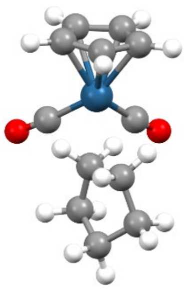

O	-0.3212	2.636515	-1.63511
H	2.79706	1.433744	-1.40489
C	-0.3907	1.689068	-0.98953
H	4.691514	-0.09607	-1.20948
H	4.925064	0.90429	0.229825
C	2.85213	0.828209	-0.49697
C	4.241857	0.203657	-0.25603
H	2.586463	1.474565	0.345568
H	1.44589	-0.54157	-1.53946
H	-1.77249	-1.05447	-2.52592
C	1.90041	-0.39058	-0.56301
Os	-0.6499	0.090732	0.04033
C	-0.3554	1.05834	1.663656
O	-0.26103	1.613607	2.665423
C	-1.88331	-1.10459	-1.45154
H	1.124987	-0.25933	0.31715
C	3.942122	-1.04104	0.594571
H	-3.3722	0.533633	-0.99606
H	2.983371	-2.2138	-0.96736
C	2.697167	-1.63661	-0.08078
H	4.772762	-1.75023	0.627369
C	-2.72893	-0.2594	-0.64004
H	3.712202	-0.74782	1.626368
H	-0.51662	-2.77379	-0.87925
C	-1.24477	-2.03024	-0.57949
H	2.114895	-2.29582	0.568336
C	-2.60782	-0.69474	0.721878
C	-1.67928	-1.79362	0.764906
H	-3.13537	-0.27195	1.566447
H	-1.3994	-2.36343	1.639633

References

1 J. D. Watson, L. D. Field and G. E. Ball, *Nat Chem*, 2022, **14**, 801–804.
2 H. S. Yu, X. He, S. L. Li and D. G. Truhlar, *Chem Sci*, 2016, **7**, 5032–5051.
3 Y. Guo, C. Riplinger, U. Becker, D. G. Liakos, Y. Minenkov, L. Cavallo and F. Neese, *Journal of Chemical Physics*, 2018, **148**, 011101.
